# Smoking Behavior and Healthcare Expenditure in the United States, 1992–2009: Panel Data Estimates

**DOI:** 10.1371/journal.pmed.1002020

**Published:** 2016-05-10

**Authors:** James Lightwood, Stanton A. Glantz

**Affiliations:** 1 School of Pharmacy, University of California, San Francisco, San Francisco, California, United States of America; 2 Center for Tobacco Control Research and Education, University of California, San Francisco, San Francisco, California, United States of America; 3 Division of Cardiology, Department of Medicine, University of California, San Francisco, San Francisco, California, United States of America; 4 Philip R. Lee Institute for Health Policy Studies, University of California, San Francisco, San Francisco, San Francisco, California, United States of America; University of Queensland, AUSTRALIA

## Abstract

**Background:**

Reductions in smoking in Arizona and California have been shown to be associated with reduced per capita healthcare expenditures in these states compared to control populations in the rest of the US. This paper extends that analysis to all states and estimates changes in healthcare expenditure attributable to changes in aggregate measures of smoking behavior in all states.

**Methods and Findings:**

State per capita healthcare expenditure is modeled as a function of current smoking prevalence, mean cigarette consumption per smoker, other demographic and economic factors, and cross-sectional time trends using a fixed effects panel data regression on annual time series data for each the 50 states and the District of Columbia for the years 1992 through 2009. We found that 1% relative reductions in current smoking prevalence and mean packs smoked per current smoker are associated with 0.118% (standard error [SE] 0.0259%, *p* < 0.001) and 0.108% (SE 0.0253%, *p* < 0.001) reductions in per capita healthcare expenditure (elasticities). The results of this study are subject to the limitations of analysis of aggregate observational data, particularly that a study of this nature that uses aggregate data and a relatively small sample size cannot, by itself, establish a causal connection between smoking behavior and healthcare costs. Historical regional variations in smoking behavior (including those due to the effects of state tobacco control programs, smoking restrictions, and differences in taxation) are associated with substantial differences in per capita healthcare expenditures across the United States. Those regions (and the states in them) that have lower smoking have substantially lower medical costs. Likewise, those that have higher smoking have higher medical costs. Sensitivity analysis confirmed that these results are robust.

**Conclusions:**

Changes in healthcare expenditure appear quickly after changes in smoking behavior. A 10% relative drop in smoking in every state is predicted to be followed by an expected $63 billion reduction (in 2012 US dollars) in healthcare expenditure the next year. State and national policies that reduce smoking should be part of short term healthcare cost containment.

## Abstract 2

James Lightwood and Stanton Glantz report on a modeling study in which health spending across US States during 1992–2009 was studied as a function of smoking prevalence, indicating that reduced smoking could yield substantial early benefits in healthcare expenditure as well as long-term improvements in health.

## Author Summary

**Why Was This Study Done?:**

- There have been many estimates of the medical costs of smoking at both the national and state levels, but these estimates do not capture the changes in health care expenditure over time that are associated with changes in smoking behavior and the effects of tobacco control programs.
- Estimates from California and Arizona have shown that medical savings accrue quickly as the prevalence and intensity of smoking decreases, when adjusted for the history of smoking reduction and tobacco control program activity in the United States.

**What Did the Researchers Do and Find?:**

- This study examined the year-to-year relationship between changes in smoking and changes in medical costs for the entire United States, taking into account differences between different states and historical national trends in smoking behavior and healthcare expenditures.
- The study found that 1% relative reductions in current smoking prevalence and mean packs smoked per current smoker are associated with 0.118% and 0.108% reductions, respectively, in per capita healthcare expenditure (elasticities).
- Historical regional variations in smoking behavior (including those due to the effects of state tobacco control programs, smoking restrictions, and differences in cigarette taxation rates) are associated with substantial differences in per capita healthcare expenditures across the United States.
- A 10% relative drop in smoking in every state is predicted to be followed by a $63 billion reduction (in 2012 US dollars) in healthcare expenditure the next year.

**What Do These Findings Mean?:**

- Changes in healthcare costs appear quickly after changes in smoking behavior.
- State and national policies that reduce smoking should be part of short term healthcare cost containment.

## Introduction

Smoking causes a wide range of diseases, including cardiovascular and pulmonary disease, complications of pregnancy, and cancers [1,2]. While the risks for some of these diseases, such as cancer, evolve over a period of years when people start and stop smoking, the risks for other diseases begin to change within days or months following changes in smoking behavior. For example, the risk of heart attack and stroke fall by about half in the first year after smoking cessation [3], and the risk of having a low birth weight infant due to smoking almost entirely disappears if a pregnant woman quits smoking during the first trimester [4]. There is a substantial literature showing that reductions in smoking behavior have substantial short and long run health benefits that reduce real per capita healthcare expenditures, beginning with reductions in cardiovascular disease, particularly heart attack and stroke [3], and respiratory disease [5]. Smoking cessation and reduction in secondhand smoke exposure in pregnant women, mothers, and children produce both very short run and long run reductions in healthcare expenditures [4,6]. The 2014 Surgeon General’s report *The Health Consequences of Smoking—50 Years of Progress* ([1], pp. 435–443) summarized 59 studies that reported immediate (often within 1 mo) 10%–20% drops in hospital admissions for acute myocardial infarction, other cardiac events, stroke, asthma, and other pulmonary events following implementation of smoke-free laws. These benefits extend to the elderly population [7], complications of pregnancy [8], and young children [8,9] and grow with time as the effects on slower-evolving diseases, such as cancer [10,11], emerge.

Previous research found that increases in per capita funding for population-based tobacco control programs in California [12,13] and Arizona [14] were associated with reductions in cigarette consumption and, in turn, with reductions in per capita healthcare expenditure in those states compared to control populations in the rest of the United States. These studies reached similar conclusions using two different aggregate measures of population smoking behavior: (1) per capita cigarette consumption in California and Arizona [12,14] and (2) smoking prevalence and cigarette consumption per smoker in California [13]. This paper extends the second approach to estimate the link between smoking behavior and healthcare expenditure for the entire United States.

## Methods

This paper estimates how much on average a 1% relative reduction in smoking prevalence in a US state reduces health costs in that state a year later. The analysis estimates this association (elasticity) while controlling for the effects of a variety of other differences between states that may produce a spurious association between reduction in smoking prevalence and reduced health expenditure, e.g., changes in population composition and other health behaviors that may also reduce health expenditure. To obtain this estimate for each state, we use a regression approach, with various refinements that take account of correlated time series. In the main and supplemental sensitivity analysis, we control—as much as possible when using state aggregated data—for the effects of other variables that may influence health care expenditure at the state level in addition to smoking (e.g., demographic factors, such as population age composition and ethnic composition; other health risk behaviors in the population, such as alcohol use; and obesity). We also control for the possible effects of unmeasured variables (e.g., cross-state cigarette purchases) on the validity of the measure of cigarette consumption per smoker in each state.

**Fig 1:**
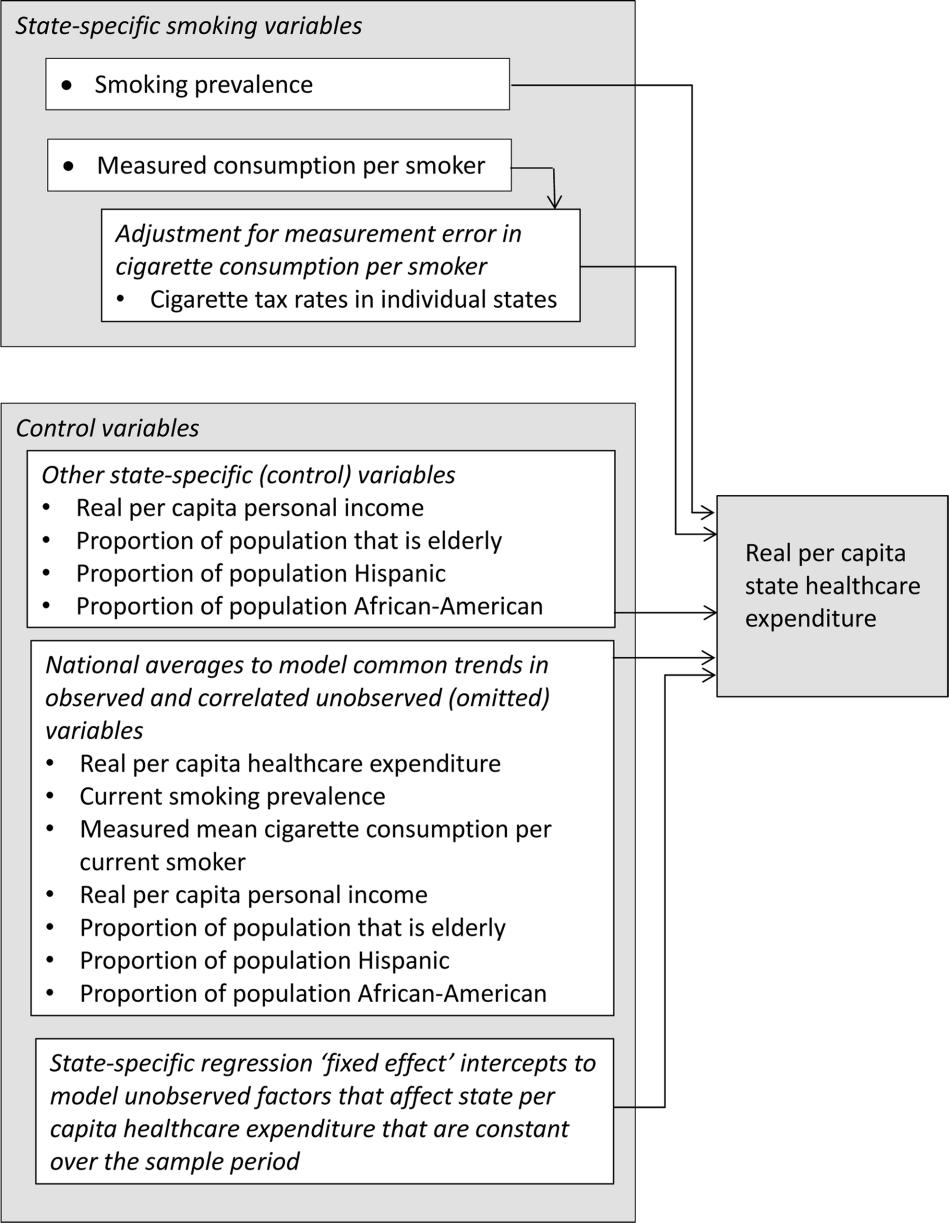
Real annual per capita state healthcare expenditure in each of the 50 states and the District of Columbia modeled as a function of smoking behavior (current smoking prevalence and mean annual cigarette consumption per smoker). Because available data on mean consumption per smoker may be contaminated with measurement error that increases over the sample period due to increasing interstate tax differentials, the individual state cigarette tax rates are included to adjust for the effects of this possible measurement error. Other state-specific control variables that might affect per capita healthcare expenditure are included. To account for long run trends in healthcare expenditure that are correlated with the observed state-specific explanatory variables as well other correlated but unobserved trends, the national averages of the dependent and explanatory variables are included in the regression. Finally, state-specific intercepts are included in the regression to model regional and state-specific factors that may affect state healthcare expenditure and that remain constant over the sample period. All the independent (explanatory) variables are lagged by 1 y.

The dependent variable in the regression model (Fig 1) is real (inflation-adjusted) annual per capita healthcare expenditure (including both public and private payers). The independent (explanatory) variables include two state-specific measures of smoking behavior (prevalence of current smoking and mean cigarette consumption per current smoker) as well as other state-specific factors that could affect healthcare expenditure (real per capita income, proportion of the population that is elderly, proportion of the population that is Hispanic, and proportion of the population that is African-American). Finally, state-specific intercepts were included in the regression to account for other factors that affect state healthcare expenditure that, while constant over time, could differ across states.

Measures of smoking behavior, the other population factors we are considering, and healthcare costs change over time unpredictably because of changes in technology, access to care, and the nature of the population itself. From a statistical perspective, that means that the underlying process is nonstationary, and we need to account for this in the analysis. To do so, we also include the national cross-sectional averages of the dependent and independent variables as independent variables in the regression equation to account for their long run trends and trends in other correlated but unobservable variables associated with per capita healthcare expenditure that vary over the sample period [15–17]. Examples of overall national trends in per capita healthcare expenditure that are difficult or impossible to measure include developments in medical technology and the economic, regulatory, legal, or legislative environment that affect access to care and therefore utilization. Including the overall national trends as independent variables means that the regression coefficients for the state-specific explanatory variables are interpreted as the effects of the variation of the state-specific variables around the overall trends included in the model. For example, the coefficient of the prevalence of current smoking in each state can be interpreted as the effect of the departure of prevalence of smoking in that state from the overall national trend in prevalence of smoking on that state’s per capita healthcare expenditure, after accounting for all the national trends included in the model.

There is also a possibility that the reported cigarette sales in a state (which we used to estimate annual per smoker cigarette consumption) might not be equal to the numbers of cigarettes smoked in a state. To adjust for possible measurement error in mean cigarette consumption per smoker, state-specific cigarette tax rates are also included in the regression model (Fig 1).

The independent variables are taken from the year before the healthcare expenditure data (i.e., lagged by 1 y), to allow for time for the independent variables to affect healthcare expenditure.

### Data

The estimated effects of smoking on healthcare costs are based on cross-sectional time series (panel) data on smoking, healthcare costs, and demographics for the 50 states and the District of Columbia (considered and referred to hereafter as 51 “states”) for the years 1992 through 2009.

#### Healthcare expenditures

The main results use the Centers for Medicare and Medicaid Services (CMS) estimates of total (public and private payer) healthcare expenditure by state of residence [18]. We chose the CMS state of residence measure because it measures healthcare expenditures consumed by residents of each state, rather than the expenditure of healthcare providers located in each state regardless of the state of the recipient. Previous research [12–14] used aggregate state data for California or Arizona compared to an aggregate population from many control states, and there was no practical or statistically significant difference in regression results using the resident- and provider-based measures. State per capita healthcare expenditure was calculated by dividing total real state expenditure by the state resident population from the US Census Bureau.

#### Smoking behavior

Prevalence of current smoking and state and federal cigarette tax data were from the Behavioral Risk Factor Surveillance System (BRFSS) provided by the Centers for Disease Control and Prevention (CDC) State Tobacco Activities Tracking and Evaluation (STATE) System [19]. State-specific per capita cigarette consumption and cigarette tax rates were from the *The Tax Burden on Tobacco* [20] provided by the CDC STATE System [19]. Cigarette consumption per smoker was calculated by dividing per capita cigarette consumption for each state’s resident population by current smoking prevalence from the US Census Bureau.

#### Demographic control variables

Total state resident population data and the proportion of state resident population age 65 y or older were from the US Census Bureau [21–23]. The proportion of the population that is Hispanic and African-American was calculated from the BRFSS survey data [24]. The proportion of the population by race and ethnicity, used for sensitivity analysis, was calculated from the BRFSS data [24] rather than census data because complete data using consistent definitions were not available from the US Census Bureau over the whole sample period, and the effects of the adjustments following the decadal census on the annual census population estimates by race and ethnicity are so large that the estimates cannot be used in regression analysis without introducing spurious results due to breaks in the model-based trends across census years. State per capita personal income was taken from the US Bureau of Economic Analysis (BEA) regional economic accounts [25].

#### Adjusting for inflation

All monetary values are expressed in year 2010 US dollars using the regional medical care (for healthcare expenditures) and regional all-item (for cigarette taxes and personal income) Consumer Price Index for All Urban Consumers (CPI-U) [26].

#### Missing data

There were up to 18 annual observations for the individual 51 states, making 918 data points. There are only 27 missing data points (2.9%) because of individual states not participating in the BRFSS in some years. All but three missing observations are due to delayed entry of 11 states into the BRFSS or a BRFSS component. Fisher’s exact test and continuity-corrected Spearman’s and Kendall’s tau-a correlation coefficients were used to evaluate the association between the presence and length of lagged state entry into BRFSS and each state’s smoking behavior and socio-demographics used in the analysis, state population, and geographic region. No statistically significant geographical or socio-demographic or economic relationships were found to explain the patterns of delayed entry among the states, so we consider the missing observations to be missing completely at random.

### Model

The regression model explains state per capita healthcare expenditure as a function of state per capita income, population age structure (proportion of the population that is elderly), proportion of the population that is African-American, proportion of the population that is Hispanic, and additional control variables that describe national trends in health care expenditure, such as changes in medical technology and the market for health care. Other variables that may affect the results were missing for some years and states, such as prevalence of insurance coverage and prevalence of other health risks (e.g., obesity and high blood pressure). A sensitivity analysis (detailed in S1 Text, Sensitivity Analyses) to determine whether inclusion of these variables would change the estimates substantially was conducted on the available observations.

Previous research compared smoking behaviors and per capita healthcare expenditures in California [12,13] and Arizona [14] to various control populations in the United States. Instead of selecting a distinct control population, this model uses the pooled common correlated effects (CCE) fixed effects estimator [15–17] on annual time series data for each of 51 cross-sectional units (the 51 states). The CCE fixed effects estimator uses the national cross-sectional averages (the arithmetic average of the 51 state-specific values for each year) of the dependent and explanatory variables to control for national trends in per capita healthcare expenditure, the other explanatory variables, and any correlated but unobservable common trends.

The model used for these national estimates has two parts (Fig 1). The details of the model appear in S1 Text (Detailed Description of the Model). The first part of the model is a first order autoregression (i.e., a regression that uses explanatory variables that are lagged one period) that models the effect of smoking behavior, adjusted for other explanatory variables, on state residential per capita healthcare expenditure. The first part of the model assumes that individual mean state cigarette consumption per smoker is observed without measurement error.

The natural logarithm of state per capita healthcare expenditure in each state is explained using the lagged natural logarithms of state smoking prevalence, mean cigarette consumption per smoker, per capita income, and several demographic variables and the lagged natural logarithms of their associated national averages across all the states. Using logarithms in this way yields regression coefficients that are interpreted as elasticities, which are dimensionless constants that give the percent change in the dependent variable associated with a 1% (relative) change in each explanatory variable. The logarithmic transformation produced better behaved residuals for individual state data than the linear specifications used in earlier work [12–14].

The second part of the model adds an adjustment for possible measurement error in individual state observations of mean cigarette consumption per smoker due to untaxed cigarette consumption induced by differences in state cigarette taxes. A state-specific model for this type of measurement error (that would use different coefficients for each of the 51 states) led to severe multicollinearity and model specification problems, so the eight BEA economic regions were chosen as the most appropriate grouping for modeling variations in the effect of the individual state-specific cigarette tax rates over time. In particular, we retained information on individual state variation in cigarette tax rates while restricting the associated coefficients’ values regionally. The BEA regions were chosen for the regional pattern of cigarette tax adjustment effects because the BEA regions reflect economically homogenous groups of states [27]. (The BEA regions are New England, Mideast, Great Lakes, Plains, Southeast, Southwest, Rocky Mountain, and Far West; the component states are listed in the first table in S1 Text.) Each individual state tax rate is assumed to have the same effect on unmeasured cigarette consumption within each BEA region, but this effect was allowed to vary across BEA regions. The implicit assumption used in choosing regional coefficients for the tax variables but not for other variables is that regional characteristics that affect unmeasured consumption (such as average size of state, distance from population centers to state borders, and cross-border commuting and other travel patterns) vary more by region than the relationship between the other explanatory variables and healthcare expenditure. This assumption was relaxed in one of the sensitivity analyses reported in S1 Text (Sensitivity Analyses).

### Sensitivity Analysis

Several sensitivity analyses were conducted to check the possibility that the estimates that attribute changes in population health to smoking are related to other risk factors than smoking (and secondhand smoke exposure). The results of these sensitivity analyses are summarized below. Detailed results appear in S1 Text (Sensitivity Analyses).

#### Other health risk factors

The prevalence of other health risk factors were measured in the BRFSS surveys (prevalence of high blood pressure and high cholesterol among respondentswho had those checked, prevalence of abusive drinking, no insurance coverage, no regular exercise, diabetes, and obesity), and these prevalence estimates were all added to the final model (Table 1), both singly and simultaneously. Inclusion of other health risk factors produced elasticity estimates that were almost identical to those shown in the final model in Table 1. In keeping with the CCE modeling strategy, these factors were added to the model as state-specific and cross-sectional trend variables. None of the variables approached statistical significance when entered into the model together or one by one (S1 Text, Sensitivity Analyses). Many states did not have observations on the other health risk factors for all years, so including these variables caused instability in the residual diagnostics. Therefore, these variables were omitted from the final analysis.

**Table 1:** Final regression results, Centers for Medicare and Medicaid Services state resident healthcare expenditure, 1992–2009.

Description of Variable	Variable	Coefficient (Elasticity)	Standard Error	*p-*Value
Prevalence of smoking	ln(*s*_*i*, *t*−1_)	0.118	0.0259	<0.001
Cigarette consumption per smoker	ln(cps_*m*, *i*, *t*−1_)	0.108	0.0253	<0.001
Per capita personal income	ln(*y*_*i*, *t−*1_)	0.224	0.0674	0.001
Percent of population age ≥ 65 y	ln(*a*_*i*, *t*−1_)	0.530	0.0936	<0.001
Percent of population Hispanic	ln(hs_*i*, *t*−1_)	0.0108	0.00763	0.156
Percent of population African-American	ln(*b*_*i*, *t*−1_)	0.0130	0.00632	0.039
Cigarette tax, New England	ln(tx_*i*, NE, *t*−1_)	0.0477	0.0103	<0.001
Cigarette tax, Mideast	ln(tx_*i*, ME, *t*−1_)	0.0203	0.0106	0.056
Cigarette tax, Great Lakes	ln(tx_*i*, GL, t−1_)	−0.00662	0.0151	0.660
Cigarette tax, Plains	ln(tx_*i*, PL, *t*−1_)	0.0358	0.0179	0.045
Cigarette tax, Southeast	ln(tx_*i*, SE, *t*−1_)	0.0190	0.0229	0.418
Cigarette tax, Southwest	ln(tx_*i*, SW, *t*−1_)	5.45 × 10^−7^	0.0248	1.00
Cigarette tax, Rocky Mountain	ln(tx_*i*, RM, *t*−1_)	−0.0108	0.0131	0.409
Cigarette tax, Far West	ln(tx_*i*, FW, *t*−1_)	0.0178	0.0312	0.568
National average per capita healthcare expenditure	ln(hr_ue, *t*−1_)	0.864	0.0959	<0.001
Principal component term*	pc3_ue, *t*−1_	−0.564	0.132	<0.001

* The “principal component term” is the third principal component of the cross-sectional average terms other than per capita healthcare expenditure. It was the only principal component that entered the regression at the 5% significance level.

#### Public policies that affect smoking behavior

Changes in smoking behavior may be correlated to other public health measures and general population awareness of healthy lifestyles, environmental health, and public policies that affect access to care. A sensitivity analysis of possible confounding by these factors was conducted by adding available time series variables that would be correlated with these factors, in the same way as was done for other health risks (S1 Text, Sensitivity Analyses). Variables describing the proportion of each state population that was covered by 100% smoke-free laws (i.e., complete smoking bans at specific venues, such as workplace, restaurants, etc.) and prevalence of lack of health insurance were added to the model in this sensitivity analysis.

#### Other factors

Consistent time series are not available for other factors that may be correlated with unmeasured changes in health risks or public health programs and policies. Perhaps the most prominent such variable is educational attainment in the population. A robustness check of the omission of this variable was conducted by studying the stability of relative state levels of educational attainment across time. Another robustness check was conducted by estimating the correlation over time between state educational attainment and a variable that should be highly correlated: state real per capita personal income.

#### Sensitivity to selection of estimation technique

Additional sensitivity analyses were conducted to evaluate the results of instrumental variable estimation for cigarette consumption per smoker by including instruments for the variables mean consumption per smoker, prevalence of cigarette smoking, per capita income, and proportion of the population age 65 y or older (S1 Text, Sensitivity Analysis). Sensitivity analyses were also conducted to account for possible correlation in healthcare expenditure between states due to unobserved factors and for other departures from standard assumptions on regression errors.

### Estimated Change in Regional Healthcare Expenditures Attributable to Smoking

The estimated elasticities in Table 1 were used to estimate the net average annual BEA regional healthcare expenditure attributable to regional cigarette smoking behavior deviations from the national average over the sample period. The unit of observation and analysis is the individual state. Therefore, the estimated changes in state expenditures were aggregated to the regional level using equal weights to calculate the aggregate results for the eight BEA economic regions. Using equal weights gives the average experience of each state in the region, which is relevant for evaluation of policy at the state level. The estimates of population-weighted changes presented in S1 Text (Effect of Weighting Scheme on Regional Healthcare Expenditures Attributable to Smoking) were used as a measure of changes in expenditure for the regional populations. The national panel regression coefficients were used for this analysis (Table 1) because eight estimates of coefficients in the model (one for each BEA region) were more reliable than 51 estimates (one for each state, with a small sample size for each regional panel regression—less than 20—for each state).

Deviations in per capita healthcare expenditures from the average national level (savings below or excess expenditures above) were calculated for each state in four steps, and then aggregated to the BEA regional level. First, for each state, the arc elasticity estimate of the deviation in state healthcare expenditure attributable to the two smoking behavior variables were calculated by multiplying the estimated elasticities of per capita healthcare for prevalence of current smoking and measured mean cigarette consumption per smoker by the average percent difference between the respective individual state and national averages of the smoking behavior variables over the sample period. The elasticities estimated in the coefficients are valid for modeling the effect of infinitesimal changes in the explanatory variables; the arc elasticity is an adjustment to account for finite differences in the data. Second, the adjustments to per capita healthcare expenditures due to state tax differentials were calculated in the same way: arc elasticities for the tax rates were calculated by multiplying the estimated elasticities of healthcare expenditure by the average percent difference between the respective individual state and national averages of the state cigarette tax variables over the sample period. Third, the net regional healthcare expenditure attributable to smoking adjusted for mismeasurement was calculated for each state by subtracting the results of the second step from the results of the first step, by state. Fourth, the excess per capita expenditures for each BEA region were calculated by taking the simple arithmetic average of each state in each respective region. Total aggregate values for each state and region were calculated by multiplying the state or regional per capita estimates by the state or regional residential populations.

As a check on the reasonableness of the results, the proportion of measured cigarette consumption per smoker due to estimated untaxed consumption was calculated. The calculation was done by dividing the healthcare expenditure due to tax differentials—and therefore attributable to mismeasurement of cigarette consumption (found in step two above)—by the average regional price of cigarettes to calculate the estimated unmeasured consumption in packs of cigarettes per capita. Estimated unmeasured consumption in packs of cigarettes per capita was then divided by the prevalence of current smokers to calculate the estimated unmeasured consumption in terms of packs per smoker. Then the estimated unmeasured consumption in terms of packs per smoker was divided by the measured mean cigarette consumption per current smoker to estimate the estimated unmeasured consumption as a proportion of measured consumption. This estimate gives the proportion of measured cigarette consumption in each region, which can be compared to survey estimates of the proportion of untaxed cigarettes consumed in the United States [28] and specific regions [29] to check the adequacy of our adjustment for measurement error in cigarette consumption and the plausibility of the resulting estimates of untaxed cigarette consumption.

Interval estimates for the excess expenditures and proportion of measured cigarette consumption that is untaxed were calculated using the covariance matrix of the elasticities (which for the logarithmic transformation is the same as the covariance matrix of coefficient matrix of the regression coefficients). The distributions of excess expenditures and proportion of unmeasured cigarette consumption were normally distributed, so formulas for the variances of functions of normal distributions were used to calculate standard errors (SEs).

Because we used the estimated elasticities to calculate the healthcare expenditure attributable to differences in smoking behavior, the estimates are independent of the sample distributions of the other variables in the model. The results can be thought of as quantifying the effects of changes in smoking behavior while holding all the other variables, such as per capita personal income and age distribution of the population, constant.

## Results

The elasticities of healthcare expenditure with respect to smoking prevalence and measured mean cigarette consumption per smoker are 0.118 (SE 0.0259, *p <* 0.001) and 0.108 (SE 0.0253, *p <* 0.001), respectively (Table 1). What these elasticities mean is that 1% relative reductions in current smoking prevalence and in packs smoked per current smoker are associated with relative reductions of 0.118% and 0.108% of per capita healthcare expenditures, respectively. For example, the average prevalence of smoking, consumption per smoker, and per capita healthcare expenditure over the sample period were 21.2%, 372 packs per year, and $6,426, respectively. A 1% relative reduction in smoking prevalence from an absolute prevalence of 21.2% to 21.0% is associated with a $7.58 reduction in per capita healthcare expenditure. Likewise, a 5% relative drop in smoking prevalence (from 21.2% to 20.1% absolute prevalence) is associated with a reduction in per capita healthcare expenditure of $37.9. A 1% relative reduction in consumption per smoker from 372 packs per year to 368 packs per year is associated with a $6.94 reduction in per capita healthcare expenditure. A 5% relative drop in consumption per smoker (from 372 packs per smoker per year to 353 packs per year) is associated with a reduction in per capita healthcare expenditure of $34.7. The *R* ^2^ statistics indicate that the regression has good explanatory power, particularly for describing variations in per capita healthcare expenditure within each state over time (Table 2).

**Table 2:** *R* ^2^ and residual statistics for final regression results.

*R* ^2^		Error Structure	
Source	Value	Statistics for Regression Residuals	Value
Within	0.914	ρ	0.940
Between	0.258	corr(*u*_*i*_, Xb)	−0.291
Total	0.495	RMSE	0.0295

ρ, proportion of regression error variance due to cross-sectional state-specific constants; corr (*u*_*i*_, Xb), correlation between linear state-specific intercept and linear score; RMSE, root-mean-square error.

These estimates of decline in per capita healthcare expenditure associated with changes in smoking behavior are counterfactual predictions that assume that all other factors other than smoking behavior remain constant. The actual observed changes in healthcare expenditure in future years will also depend on additional state-specific variables such as per capita income and age structure of the population, in addition to their evolution via common trends across states.

### Sensitivity Analyses

None of the sensitivity analyses for omitted variables produced a statistically significant or even barely noticeable change in the regression coefficients of the estimated model (S1 Text, Sensitivity Analyses). The other health risk factors and policy variables do not seem to be highly correlated, at least on a population level. In other conditions, there are significant state and regional differences—and therefore significant correlation between the variables and smoking behavior—at any one point in time, but there is little variation between states over time. For example, in the case of obesity, at any one point in time, some states with high smoking prevalence have a higher than average prevalence of obesity. However, the prevalence of obesity in all states is increasing at approximately the same rate over time, albeit from different starting levels. For this reason, state-level variations in obesity in a particular year do not confound state-level variations in smoking behavior over time. The robustness analysis on education showed that the correlation between states in educational attainment over time was high, particularly for the prevalence of bachelor degrees in the population over time. However, state prevalence of both high school completion and bachelor degrees was highly correlated over time with state real per capita personal income; therefore, we believe the possible direct effects of education on health care expenditure or indirect effects through correlation with smoking behavior are accounted for in the per capita income variable.

The results of the sensitivity analysis on instrumental variables did not produce evidence of serious bias produced by problems with the instruments used for cigarette consumption per smoker, except for proportion of the population age 65 y or over (S1 Text, Sensitivity Analyses). When the proportion of the population that was elderly was instrumented, the coefficient of that variable was reduced by about half, but the change in the coefficient was not statistically significantly different from that presented in Table 1. There were no substantial changes in the coefficients of the other variables. There was no trend in the coefficient estimates as a function of factors that could produce bias, such as the strength of autocorrelation in the regression residuals, and the SEs of the estimates presented in Table 1 were consistent with the point coefficient estimates of the sensitivity analysis.

### Estimated Change in Regional Healthcare Expenditures Attributable to Smoking

Without adjustment for mismeasurement of cigarette consumption per smoker, the Far West region has the largest estimated savings in annual per capita healthcare expenditure associated with departures of its smoking behavior from the national average: $210 (SE $45.5); the Southeast region has the largest excess expenditure: $154 (SE $30.7) (Table 3).

**Table 3:** Average excess expenditures associated with departures of regional smoking behavior and cigarette consumption from national average, 1992–2009.

Average Excess Expenditure	BEA Region							
	New England	Mideast	Great Lakes	Plains	Southeast	Southwest	Rocky Mountain	Far West
**Attributable to prevalence of smoking**								
Mean	−37.0	−34.8	62.5	−21.7	66.4	−6.54	−119	−34.5
SE	6.80	7.65	13.8	4.76	14.6	1.45	26.1	7.62
**Attributable to mean cigarette consumption per smoker**								
Mean	42.1	−68.6	−19.1	10.9	87.8	−134	−16.7	−175
SE	9.86	16.0	4.50	2.55	20.5	31.4	3.90	41.1
**Attributable to differences in smoking behavior: prevalence and mean cigarette consumption per smoker**								
Mean	5.30	−103	43.4	−10.7	154	−141	−135	−210
SE	9.00	21.0	12.1	4.09	30.7	32.1	28.3	45.5
**Attributable to state tax differential effects**								
Mean	98.5	30.0	−2.65	−34.0	−59.9	0.00104	14.6	28.0
SE	21.5	15.8	6.01	17.0	74.2	6.29	17.8	49.6
**Implied proportional difference between measured and estimated true cigarette consumption per smoker (proportion)**								
Mean	0.416	0.163	−0.0165	−0.141	−0.236	0.00000317	0.0791	0.164
SE	0.0906	0.0860	0.0374	0.0704	0.292	0.0192	0.0962	0.290
**Total attributable to differences in smoking behavior including state tax differential effects**								
Mean	104	−73.4	40.7	−44.8	94.4	−141	−121	−182
SE	25.4	25.4	11.5	17.5	90.2	34.0	32.7	51.7
**Total regional difference, including state tax differential effects (millions of 2010 US dollars)**								
Mean	1,500	−3,530	1,890	−910	7,330	−5,210	−1,310	−9,470
SE	370	1,220	367	356	7,010	1,260	355	2,690

Data are given as 2010 US dollars per capita unless otherwise indicated. Negative dollar amounts indicate savings compared to national average smoking behavior; positive dollar amounts indicate excess expenditures compared to national average smoking behavior. Negative proportions indicate that estimated true consumption is less than measured consumption; positive proportions indicate that estimated true consumption is less than measured consumption.

After adjustment for state tax differentials, the Far West still has the largest total estimated annual per capita savings, $182 (SE $51.7), but the New England region now has the largest excess per capita expenditure, $104 (SE $25.4); the Southeast has the next largest, $94.4 (SE $90.2) (Table 3). Total annual estimated expenditure per year due to the differences between regional and national smoking behavior ranges from a savings of $9,470 million (SE $2,690 million) in the Far West to a total excess expenditure of $7,330 million (SE $7.010 million) in the Southeast region (Table 3).

The difference between measured and estimated true cigarette consumption per smoker was less than 20% for all BEA regions except the Southeast, where estimated true consumption was 23.6% (SE 29.2%) less than measured consumption, and New England, where estimated true consumption was 41.6% (SE 9.06%) higher than measured (Table 3). These estimates are similar to estimates from survey data collected by examining the source of cigarette packs in different states in 2009 and 2010 [28]. The model’s statewide estimates of the proportion of cigarette consumption that is untaxed track survey estimates [29] for major urban centers in the Mideast and New England reasonably well (Table 4). The comparisons are complicated by two factors: the difference in areas in the regions covered and that the survey estimates provide only ranges based on modeling assumptions. For example, untaxed consumption may be unusually high in New York City due to high local cigarette tax rates and may be higher there than on average in other areas of New York state. See S1 Text (State-Specific Healthcare Expenditures Attributable to Smoking) for population-weighted regional and individual state estimates of excess expenditure associated with smoking behavior.

**Table 4:** Survey and model estimates of percent of cigarette consumption that is untaxed.

Survey Estimates [29]			Model Estimates			
Metropolitan Area	Range		Area	Point Estimate	95% Confidence Interval	
	Low	High			Low	High
New York City	47.9%	49.9%	New York State	20.1%	8.02%	32.2%
Boston	36.8%	38.4%	Massachusetts	34.2%	27.5%	40.9%
Providence	29.6%	55.4%	Rhode Island	35.3%	28.1%	41.9%
Philadelphia	1.2%	1.3%	Pennsylvania	4.9%	2.8%	7.0%
District of Columbia	29.0%	59.9%	District of Columbia	13.1%	4.7%	21.5%

Survey estimates provide ranges based on modeling assumptions, rather than 95% confidence intervals.

## Discussion

Our estimates provide strong evidence that reducing smoking prevalence and cigarette consumption per smoker are rapidly followed by lower healthcare expenditure. The model is dynamic and predicts per capita healthcare expenditures in the current year as a function of smoking behavior in the previous year. For example, 1% relative reductions in current smoking prevalence and mean cigarette consumption per smoker in one year are associated with a reduction in per capita healthcare expenditure in the next year of 0.118% + 0.108% = 0.226% (SE 0.0363%), with all other factors including common trends held equal. In 2012, total healthcare expenditures in the US were $2.8 trillion [30]; our results suggest that, holding other common trends and factors affecting health care expenditures constant, a 10% relative drop in smoking prevalence (about a 2.2% absolute drop) combined with a 10% relative drop in consumption per remaining smoker (about 37 fewer packs/year) would be followed in the next year by a $63 billion reduction in healthcare expenditure (in 2012 dollars).

These are short run 1- to 2-y predictions, and while they indicate that the effects of changes in smoking on healthcare expenditure begin to appear quickly, they do not imply that all changes in the costs and savings of smoking in the population are immediate. If all states reduce their prevalence of smoking and cigarette consumption per smoker, then the corresponding common trends will gradually change over time. The elasticity of the common trend for the prevalence of smoking (from the model estimated with all cross-sectional averages entered as separate variables, rather than using principal components) is relatively small and not statistically significant (−0.0545, SE 0.0581, *p =* 0.348), so it is unlikely to play a large role in longer run predictions. The elasticity of the common trend for cigarette consumption per smoker (−0.255, SE 0.0488, *p <* 0.001) is not small relative to the state-specific cigarette consumption per smoker variable. Over the longer run, changes in both smoking behavior variables will change the age structure of the population and trends in changes in healthcare expenditures related to the prevalence of elderly people in the population. Therefore, longer run predictions require a formal out-of-sample forecast study. The short run illustrative predictions presented here also assume the continuation of historical aggregate trends that have been associated with tobacco control policies, such as the declines in exposure to secondhand smoke and in prevalence of smoking during pregnancy.

These estimates are consistent with previous research on healthcare expenditures attributable to cigarette smoking in California [12,13] and Arizona [14]. The previous research used the aggregate population in control states to account for common trends in healthcare expenditure, while the present study used the cross-sectional average expenditure across states. The regression specifications also differ. In the previous research, specification searches were used to determine the best regression model to use to estimate the effects of smoking in California and Arizona versus the control states. Similar specification searches for each of the 51 cross-sectional units (i.e., states) in the present study were not feasible, and variables that are probably irrelevant for California and Arizona were left in the specification because they are required to be in the model for other states. However, inclusion of irrelevant variables for a state will not bias the estimated elasticities and permits estimating an average effect across all states with a simple panel regression specification.

This analysis uses aggregate measures of population characteristics to estimate the relationships between smoking behavior variables and per capita healthcare expenditures. The elasticity estimates are not directly comparable to estimates of the economic burden of cigarette smoking using cross-sectional data on individuals in national health surveys [31]. Those estimates use data on individuals to calculate the healthcare expenditure attributable to cigarette consumption in individual current smokers or ever-smokers, contrasted to individual non-smokers or never-smokers, respectively. Therefore, the expenditure estimates in the present study should not be interpreted as healthcare costs arising in, or due to, individual smokers or any specific individuals in the population. These estimates reflect all the healthcare expenditures associated with smoking that arise in a population, which include short and long term indirect effects on smokers and short and long term effects of second- and third-hand [32] smoking exposure in non-smokers. However, previously published aggregate estimates for California [13] that are similar to those presented here are somewhat larger than, but consistent with, cross-sectional estimates for that state using individual survey data [33], and the difference between these estimates is comparable to variation among different published cross-sectional estimates based on individual data [6,34,35].

Our estimates do avoid some problems of estimates based on cross-sectional data. An example is the “quitting sick” effect, which imputes expenditure savings to smokers who quit smoking after being diagnosed with a serious chronic tobacco-related disease, such as lung cancer or cardiovascular disease. The expected expenditure savings from quitting by a smoker who remains well will not be realized in those who quit sick because expensive and irreversible health effects of smoking have already occurred. The quitting sick effect is a consequence of incorrectly imputing missing information (the unobservable health status of the smoker at the time of cessation) that is not present in cross-sectional data. This study uses longitudinal data on measures of smoking behavior and healthcare expenditures on large populations and therefore is not subject to quitting sick effects because the excess health care costs of those who quit sick will be included in a state’s total aggregate healthcare expenditure data along with the reduction in prevalence that occurs when the reduction in smoking of comparable people is recorded in surveys that represent the population of that state. It should be noted that some estimates of the health burden of cigarette smoking that account for quitting sick and other problems with estimates based on cross-sectional data find a higher burden of smoking-related disease and therefore higher smoking-attributable expenditures than most published cross-sectional estimates [36–40].

The estimates presented here cannot be used to reliably estimate the change in healthcare expenditure associated with complete elimination of cigarette consumption because the estimated elasticities apply only to modest variation around the status quo, but they do capture expenditures attributable to cigarette smoking in a large population that are difficult to measure from national health surveys (such as the effects of second- and third-hand smoke exposure, and long term effects of developmental problems from premature birth and low birth weight or asthma contracted during childhood, attributable to parental cigarette smoking).

Our methods may suffer from spurious regressions and attribute non-smoking public health factors that are correlated with smoking behavior to the smoking behavior. Specifically, this research does not estimate a smoking attributable fraction of healthcare costs for each state that corresponds to a measure that can be derived from individual survey data. Rather, it estimates the average national effect of variations in aggregate-level state-specific smoking behavior variables around the national trend in those variables on variations in state-specific real per capita healthcare expenditure around its national trend.

### Limitations

The results of this study are subject to the limitations of analysis of aggregate observational data. A study of this nature that uses aggregate data and a relatively small sample size cannot, by itself, establish a causal connection between smoking behavior and healthcare costs, and that is not the goal of this study. Rather, this study should be evaluated in the context of the existing body of research that has already established that the relationship between smoking behavior and healthcare costs is causal using a variety of study designs [41–45].

These estimates do not address the issue of whether, over the whole life cycle, a population without any cigarette smoking would have higher healthcare expenditures due to longer lived non-smokers. Forecasting the very long run effects of reductions in smoking over the life cycle in a US population would require the construction of a model to forecast the eventual changes in the age structure of the population and resulting changes in per capita healthcare expenditures as a function of smoking behavior.

### Conclusions

Lower smoking prevalence and cigarette consumption per smoker are associated with lower per capita healthcare expenditures. Historical regional variations in smoking behavior (including those due to the effects of state tobacco control programs, smoking restrictions, and differences in taxation) are associated with substantial differences in per capita healthcare expenditures across the United States. Those regions (and the states in them) that have implemented public policies to reduce smoking have substantially lower medical costs. Likewise, those that have failed to implement tobacco control policies have higher medical costs. Changes in healthcare costs begin to be observed quickly after changes in smoking behavior. State and national policies that reduce smoking should be part of short term healthcare cost containment.

## Supporting Information

S1 Text Model estimation, additional detailed results, and sensitivity analysis. (PDF)

## Abbreviations

BEA: US Bureau of Economic Analysis
BRFSS: Behavioral Risk Factor Surveillance System
CCE: common correlated effects
CMS: Centers for Medicare and Medicaid Services
SE: standard error
