# Supplementary material for: Smoking Behavior and Healthcare Expenditure in the United States, 1992–2009: Panel Data Estimates
Source: PLoS Med. 2016 May 10;13(5):e1002020. doi: 10.1371/journal.pmed.1002020 (PMC4862673; doi:10.1371/journal.pmed.1002020)
Supplement: S1 Text — (PDF) [file pmed.1002020.s001.pdf]

SUPPLEMENTAL TEXT S1

MODEL ESTIMATION, ADDITIONAL DETAILED RESULTS, AND SENSITIVITY ANALYSIS

Smoking Behavior and Healthcare Expenditure in the United States, 1992-2009: Panel Data Estimates

James Lightwood*

Stanton A. Glantz**

* School of Pharmacy and Center for Tobacco Control Research and Education, University of California, San Francisco, California, United States of America
** Department of Medicine (Cardiology), Center for Tobacco Control Research and Education, and Philip R. Lee Institute for Health Policy Studies, University of California, San Francisco, California, United States of America

TABLE OF CONTENTS

DETAILED DESCRIPTION OF THE MODEL 4

Relationship Between Smoking Behavior and per capita Healthcare Expenditures 4

Accounting for Nonstationarity 9

Correction for Possible State Cigarette Consumption Measurement Error 10

Estimated Regression Model 13

MODEL ESTIMATION 15

Statistical Calculations 18

Development of the Final Model 19

ADDITIONAL DETAILED RESULTS 20

Model Estimates 20

Demographic Results 22

SENSITIVITY ANALYSES 22

Alternative Estimators 24

Bootstrap Standard Errors 25

Inclusion of Time Trends 25

Sensitivity to Choice of Cross-Sectional Units 26

Estimation with Heterogeneous Regional Slope Coefficients 28

Instrumental Variables Estimator for Measurement Error in Prevalence of Current Smoking . 32

Instrumental Variables Estimation 34

First stage estimates and instrumental variables diagnostics for Final Regression Results in the Main text (Table 1) 35

Instrumental Variables Estimator Sensitivity Analysis 36

Effect of Weighting Scheme on Regional Healthcare Expenditures Attributable to Smoking 40

STATE-SPECIFIC HEALTHCARE EXPENDITURES ATTRIBUTABLE TO SMOKING 41

COMPARISON TO PREVIOUS RESULTS 42

VARIATION IN PREVALENCE OF SMOKING AND CONSUMPTION PER SMOKER..... 43

REFERENCES 45

DETAILED DESCRIPTION OF THE MODEL

Relationship Between Smoking Behavior and per capita Healthcare Expenditures

The first part of the model estimates the natural logarithm of healthcare expenditure assuming that the true value for mean cigarette consumption is observable. The dependent variable, the natural logarithm of state per capita healthcare expenditure is explained using the natural logarithms of several explanatory variables. Using logarithms in this way yields regression coefficients that are interpreted as elasticities, which are dimensionless constants that give the percent change in the dependent variable associated with a one percent (relative) change in each explanatory variable. The logarithmic transformation produced better behaved residuals using individual state data than the linear specifications used in our earlier work [1,2,3]. The main equation is

$$\ln(h_{i,t}) = \alpha_0 + \alpha_{0,i} + \alpha_1 \ln(s_{i,t-1}) + \alpha_2 \ln(cps_{i,t-1}) + \alpha_3 \ln(y_{i,t-1}) + \alpha_4 \ln(a_{i,t-1}) + \alpha_5 \ln(hs_{i,t-1}) + \alpha_6 \ln(b_{i,t-1})$$
$$+\alpha_7 \ln(h_{ue,t-1}) + \alpha_8 \ln(s_{ue,t-1}) + \alpha_9 \ln(cps_{ue,t-1}) + \alpha_{10} \ln(y_{ue,t-1}) + \alpha_{11} \ln(a_{ue,t-1}) + \alpha_{12} \ln(hs_{ue,t-1})$$
$$+\alpha_{13} \ln(b_{ue,t-1}) + \varepsilon_{1,i,t} \quad (1)$$

where

$\ln(h_{i,t})$ is the natural logarithm of annual real Centers for Medicare and Medicaid Services (CMS) resident per capita healthcare expenditure in state *i* in year *t*, in thousands of 2010 dollars,

$\ln(s_{i,t-1})$ is the natural logarithm of prevalence of current smoking in state *i* in year *t*-1, in percentage points,

$\ln(cps_{i,t-1})$ is the natural logarithm of annual mean cigarette consumption per current smoker in state *i* in year *t*-1, in 100 packs/year per smoker,

$\ln(y_{i,t-1})$ is the natural logarithm of annual real per capita personal income in state *i* in year *t*-1, in ten thousands of 2010 dollars,

$\ln(a_{i,t-1})$ is the natural logarithm of proportion of the population age 65 years and over in state *i* in year *t*-1, in percentage points,

$\ln(hs_{i,t-1})$ is the natural logarithm of proportion of the population Hispanic in state *i* in year *t*-1, in percentage points,

$\ln(b_{i,t-1})$ is the natural logarithm of proportion of the population African-American in state *i* in year *t*-1, in percentage points,

$\ln(h_{ue,\, t-1})$ is the natural logarithm of real annual national cross sectional average of per capita healthcare expenditures in year *t*-1, in thousands of 2010 dollars,

$\ln(s_{ue,t-1})$ is the natural logarithm of national cross sectional average of prevalence of current smoking in year *t*-1, in percentage points,

$\ln(cps_{ue,t-1})$ is the natural logarithm of annual national cross sectional mean of cigarette consumption per current smoker in year *t*-1, in 100 packs/year,

$\ln(cps_{ue,t-1})$ is the natural logarithm of real annual national cross-sectional average of per capita personal income in year *t*-1, in ten thousands of 2010 dollars,

$\ln(a_{ue,t-1})$ is the natural logarithm of national cross sectional average of proportion of the population age 65 years and over in year *t*-1, in percentage points,

$\ln(hs_{ue,t-1})$ is the natural logarithm of national cross sectional average of proportion of the population Hispanic in year *t*-1, in percentage points,

$\ln(b_{ue,t-1})$ is the natural logarithm of national cross sectional average of proportion of the population African-American in year *t*-1, in percentage points,

$\varepsilon_{1,i,t}$ is the error term for state *i*, in year *t*,

i is the index for 50 states and District of Columbia (51 cross-sections, the units of analysis),

t is the time index, t = 1992 to 2009 (up to 18 annual observations due to missing observations for some states in some years).

The Independent Variables

Equation 1 explains per capita personal healthcare expenditures in state *i* in year *t* as a function of three groups of effects.

The first group contains a common constant, α_0, and a set of state-specific constants, $\alpha_{0,i}$. The state-specific constants control for state-specific explanatory variables that differ between states, but remain constant over the sample period within each state.

The second group ($\ln(s_{i,t-1})$ $\ln(cps_{i,t-1})$, $\ln(y_{i,t-1})$, $\ln(a_{i,t-1})$, $\ln(hs_{i,t-1})$, $\ln(b_{i,t-1})$) contains observable state-specific variables that explain variations in per capita healthcare expenditure around national trends. The coefficients associated with the second group represent the effects of departures of those state-specific variables from overall national trends in the explanatory variables and correlated unobservable trends. Several variables had very small and statistically insignificant coefficients (using the conventional 5% significance level) and had an almost unnoticeable effect the other coefficient estimates (e.g., such as state proportion of resident and smoking population that is male and population coverage of local and state of smoke-free laws). These variables were omitted from the basic model specification.

The third group ($\ln(h_{ue,t})$, $\ln(s_{ue,t})$ $\ln(cps_{ue,t-1})$, $\ln(y_{ue,t-1})$, $\ln(a_{ue,t-1})$, $\ln(hs_{ue,t-1})$ $\ln(b_{ue,\,t-1})$) contains national cross sectional averages of the state-specific explanatory variables and of per capita health care expenditure (the dependent variable) which control for common national trends in these observable and other unobservable but correlated trends associated with per capita healthcare expenditure that vary over the sample period [4,5,6]. Each cross-sectional average is one time series with the same number of observations as there are years in the sample. Many weighting schemes can be used for the national cross sectional averages ($\ln(h_{ue,t})$, $\ln(s_{ue,t})$ $\ln(cps_{ue,t-1})$, $\ln(y_{ue,t-1})$, $\ln(a_{ue,t-1})$, $\ln(hs_{ue,t-1})$ $\ln(b_{ue,\,t-1})$), all of which produce identical asymptotic results. This study followed the most common convention and used the simple arithmetic means of the state level data [4,5,6]. Using the simple arithmetic mean to represent cross-sectional averages is also most appropriate when the individual states are the unit of analysis using aggregate data. For example, the cross-sectional average of per capita healthcare expenditure, $(\ln(h_{ue,t-1}))$ for 1995 is the simple arithmetic average of each state's annual per capita healthcare expenditure in the year 1995.

Past research on the effect of the second group of state-specific variables using cross sectional data suggests that the coefficients for prevalence of current smoking, $\ln(s_{i,\,t-1})$, cigarette consumption per smoker, $\ln(cps_{i,t-1})$ **,** should be positive [1,2,3], though they may be negative in studies with longitudinal data in populations where increased longevity due to lower levels of smoking increases life expectancy enough to increase average healthcare per capita expenditures [7]. Past research suggests that the coefficients of the real personal per capita income $\ln(y_{i,t-1})$ should be positive [8,9]. Estimates of the effect of age structure of the population, $\ln(a_{i,t-1})$, measured as the proportion of the population age 65 years and over, has

produced mixed results [8], though is generally assumed to be positive because per person annual healthcare expenditures rise with age in cross-sectional analyses.

The proportions of population Hispanic, $\ln(hs_{i,t-1})$ and African-American, $\ln(b_{i,t-1})$ were included to model the effect of having a high proportion of these groups because Hispanics and African-Americans may have different smoking behavior [10], health status, access and utilization of health care [11,12,13,14] than other subpopulations. The net effect of prevalence of Hispanic and African-American race/ethnicity on per capita health care expenditure is ambiguous because their effects operate through several channels, including different health risk behaviors, income, social customs affecting utilization and access to care.

The third group of variables, the national cross-sectional averages ($\ln(h_{ue,t})$, $\ln(s_{ue,t})$ $\ln(cps_{ue,t-1})$, $\ln(y_{ue,t-1})$, $\ln(a_{ue,t-1})$, $\ln(hs_{ue,t-1})$, $\ln(b_{ue,\,t-1})$) reflects common trends over time of unobservable factors associated with national health care expenditures. Examples of common trends that may be correlated with per capita health care expenditure, $h_{ue,t-1}$, are national trends in health care technology, health care insurance coverage and access to care, and standards of care. The coefficient of average per capita healthcare expenditure, $\ln(h_{ue,t-1})$, should be positive because there has been a strong secular upward trend in per capita expenditures in all states independent of smoking behavior. The cross sectional averages of prevalence of smoking, $s_{ue,t-1}$, and mean consumption per smoker, $cps_{ue,\,t-1}$, may be correlated with national trends in unobservable characteristics of smoking behavior (e.g., prevalence of non-daily smoking) or prevalence of second and third hand smoking for which annual state and regional level data are not available over the whole sample period. These cross sectional averages may also represent

the net effect of a number of unobservable common trends that are correlated with the observable explanatory variables, so their signs are difficult to predict.

Accounting for Nonstationarity

There is strong evidence that several variables, including state-specific per capita healthcare expenditure and per capita state personal income, are non-stationary with auto-regressive unit roots or very nearly non-stationary in the sample [1,3,9,15]. The interpretation of the regression coefficients in a panel regression with nonstationary variables depends on the independence of the regression errors over cross-sectional units (that is, over the states). In terms of equation 1, cross sectional dependence means that $\varepsilon_{1,j,t}$ and $\varepsilon_{1,,k,t}$ are correlated when $j \neq k$.

There are three possible patterns in the regression coefficients and correlation in the regression errors: (1) If the regression coefficients are constant across states and the regression error terms are independent across states, the regression coefficients are consistent estimates describing the stable relationship between the dependent and explanatory variables regardless of whether the regression error term, $\varepsilon_{1,i,t}$, is stationary (that is, there is co-integrating relationship between the dependent and explanatory variables) or non-stationary (that is, there is no co-integrating relationship). (2) If the regression coefficients differ across states and the regression error terms are correlated between the states, the estimated regression slope coefficients may not have any particular relationship to the true regression parameters if the regression errors are non-stationary (that is, if there is no cointegrating relationship). (3) If the regression coefficients vary across states and there is dependence between the error terms across the states and the residuals are stationary (that is, there is a cointegrating relationship), the coefficients can be interpreted as weighted averages of the true state-specific coefficients [4,16].

In this study there is some ambiguous evidence of correlation between regression error-terms and a sensitivity analysis produced some evidence that the slope coefficients of some variables vary across states. For that reason it is important to examine the time series behavior of the regression residuals and explore any possible effect of nonstationarity in the regression residuals (the consequence of there being no cointegrating relationship) on the coefficient estimates and their interpretation. As the results below show, possible non-stationarity in the regression residuals, and correlation of regression errors across states had little effect on the results.

Correction for Possible State Cigarette Consumption Measurement Error

The second part of the model is an adjustment for observed mean cigarette consumption per smoker in individual states that corrects for possible mismeasurement of state cigarette consumption due to tax avoidance. If a state increases its cigarette tax, other factors held constant (including other states' cigarette taxes), the level of true cigarette consumption per smoker might increase relative to measured consumption due to an increased incentive to avoid paying the tax in that state. The data used to calculate mean cigarette consumption per smoker is based on state cigarette tax records [17,18] that do not account for untaxed cigarette consumption due to cross border sales (e.g., internet sales, smuggling and casual tax avoidance by commuters and travelers), sales from military bases and Native American reservations, or counterfeit cigarettes. True mean cigarette consumption per smoker may differ from the consumption measured by individual state tax records. The differences between state cigarette excise tax rates, and therefore interstate tax differentials, were relatively stable from 1992-1998 but became more variable after 1999 [19,20]. Therefore, state-specific tax rates were included in the equation to adjust the for unmeasured mean cigarette consumption due to changes in state and federal tax

rates that change interstate tax differential and therefore could change incentives for untaxed consumption over the sample period.

Several specifications for the tax-adjustment for untaxed cross-border cigarette consumption were estimated: a model of net inflows and outflow of untaxed cigarettes due to positive and negative interstate tax differentials, models of intra-and inter-regional flow of untaxed cigarettes, separate adjustments for short and long distance movement of untaxed cigarettes across state borders and from internet export sales states in the Southeast (Kentucky, North Carolina, and Virginia), None of the alternative approaches produced practically or statistically significant changes in the coefficients that described the relationship between healthcare expenditures and smoking behavior and demographic variables.

Allowing for different coefficients for all 51 states in the model led to severe multicolinearity and model specification problems. The adjustment that performed best in terms of standard regression diagnostics was the simplest: the natural logarithm of individual state cigarette tax rates where the coefficients of the state tax were allowed to vary by BEA economic region. The eight Bureau of Economic Analysis (BEA) Economic Regions were chosen as the most appropriate grouping for modeling variations in the effect of state cigarette tax rates because the BEA regions reflect economically homogenous groups of states [21]. (The BEA regions are New England [NE], Mideast [ME], Great Lakes [GL], Plains [PL], Southeast [SE], Southwest [SW], Rocky Mountains [RM], and Far West [FW]; Supplemental Table S1.) Individual state tax rate is assumed to have the same effect on unmeasured cigarette consumption within each BEA region, but this effect was allowed to vary across BEA regions. This specification allows the effect of cigarette tax rates on unmeasured consumption to vary by region. For example, the effect of the same tax rate in a state in New England may have a

different effect on untaxed consumption than it would in the Far West. The implicit assumption used in choosing regional coefficients for the tax variables but not for other variables, is that regional characteristics that affect unmeasured consumption (such as average size of state, distance of population centers to state borders, cross-border commuting and other travel patterns) vary more by region than the relationship between the other explanatory variables and healthcare expenditure.

The measurement adjustment for mean cigarette consumption per smoker is

$$\ln(cps_{i,t}) = \ln(cps_{m,i,t}) + \beta_0 + \beta_{0,i} + \sum\nolimits_{r=1}^{8} \beta_{1,r} \ln(tx_{i\in r,t}) + \varepsilon_{2,i,t}\,, \qquad (2)$$

where

$\ln(cps_{m,i,t})$ is the logarithm of the measured mean cigarette consumption per current smoker in state *i* in year t from state tax records, in 100 packs in year *t*,

$\ln(tx_{i\in r,t})$ is the natural logarithm of the cigarette tax in state *i* in region r in year *t*, in 2010 dollars per pack, and zero for state *i* that is not in region *r*,

r is the index for states in BEA region *r*, *r*= 1, …, 8

$\varepsilon_{2,i,t}$ is a stationary error term for state *i* in year *t*.

Equation 2 models the effect of variation in the cigarette tax rate in state *i* on the difference between the measured and true mean cigarette consumption in state *i*, controlling for the tax rates in all other states. An increase in cigarette taxes should be associated with an increase in the difference between true and measured cigarette consumption due to increased incentives for consuming untaxed cigarettes. However, state tax rates may also affect state smoking behavior in ways that are not captured in the two dimensional measure used in this study (current smoking prevalence and mean cigarette consumption per smoker). For example, an increase in the real tax rate may shift the distribution of cigarette consumption by current

smokers downward in a way not captured by mean consumption per smoker such as changing the distribution of daily versus non-daily smoking , which may, in turn, affect healthcare expenditure. While the tax elasticities (the coefficients $\beta_{1,r}$) are expected to be positive if the main association in the sample is between an increase in state cigarette taxes and untaxed in-state consumption, they could be negative if an offsetting effect is present on the distribution of cigarette consumption per smoker that is not captured in prevalence of smoking or mean cigarette consumption per current smoker.

Estimated Regression Model

Substituting equation 2 into equation 1 produces

$$\ln(h_{i,t}) = (\alpha_0 + \alpha_2\beta_0) + (\alpha_{0,i} + \alpha_2\beta_{0,i}) + \alpha_1 \ln(s_{i,t-1}) + \alpha_2 \ln(cps_{m,i,t-1})$$
$$+ \alpha_3 \ln(y_{i,t-1}) + \alpha_4 \ln(a_{i,\,t-1}) + \alpha_5 \ln(hs_{i,t-1}) + \alpha_6 \ln(b_{i,t-1})$$
$$+ \alpha_7 \ln(h_{ue,\,t-1}) + \alpha_8 \ln(s_{ue,t-1}) + \alpha_9 \ln(cps_{ue,t-1}) + \alpha_{10} \ln(y_{ue,t-1}) + \alpha_{11} \ln(a_{ue,t-1}) + \alpha_{12} \ln(hs_{ue,t-1}) + \alpha_{13} \ln(b_{ue,t-1})$$
$$+ \alpha_2 (\textstyle\sum_{r=1}^{8} \beta_{1,r} \ln(tx_{i\in r,,t-1})) + (\varepsilon_{1,i,t} + \alpha_2\varepsilon_{2,i,t-1}) \qquad (3)$$

Collecting terms, the equation to estimate is

$$\ln(h_{r,\,t}) = \gamma_0 + \gamma_{0,r} + \gamma_1 \ln(s_{r,t-1}) + \gamma_2 \ln(cps_{m,i,,t-1}) + \gamma_3 \ln(y_{i,t-1}) + \gamma_4 \ln(a_{i,t-1}) + \gamma_5 \ln(hs_{i,t-1}) + \gamma_6 \ln(b_{i,t-1})$$
$$+ \gamma_7 \ln(h_{ue,\,t-1}) + \gamma_8 \ln(s_{ue,t-1}) + \gamma_9 \ln(cps_{ue,t-1}) + \gamma_{10} \ln(y_{ue,t-1}) + \gamma_{11} \ln(a_{ue,t-1}) + \gamma_{12} \ln(hs_{ue,t-1}) + \gamma_{13} \ln(b_{ue,t-1})$$
$$+ \gamma_{14} \ln(tx_{i\in NE,t-1}) + \gamma_{15} \ln(tx_{i\in ME,t-1}) + \gamma_{16} \ln(tx_{i\in GL,t-1}) + \gamma_{17} \ln(tx_{i\in PL,t-1}) + \gamma_{18} \ln(tx_{i\in SE,t-1}) + \gamma_{19} \ln(tx_{i\in TE,t-1})$$
$$+ \gamma_{20} \ln(tx_{i\in SW,t-1}) + \gamma_{21} \ln(tx_{i\in RM,t-1}) + \gamma_{22} \ln(tx_{i\in FW,t-1}) + \upsilon_{i,t} \qquad (4)$$

where

$$\upsilon_{i,t} = \varepsilon_{1,i,t} + \alpha_2\varepsilon_{2,i,t-1}.$$

As noted earlier, because logarithmic transformations are applied to the dependent and explanatory variables, the coefficients are interpreted as elasticities.

Correlation between the explanatory variables and the regression error term (which violates the assumptions for standard regression analysis) may occur in the model and require special estimation techniques for two reasons. The first reason is correlation between explanatory variables and the regression error due to measurement error. Measurement error may occur for mean consumption per smoker and is the reason for the measurement error equation described above. Cigarette manufacturers and wholesalers may anticipate state tax increases, which often take effect with a considerable lag after passage, and act strategically by stockpiling cigarettes for sale shortly before the tax increase. Higher tax levels may encourage long range inter-regional sales by mail and internet, and other tax avoidance methods that may be imperfectly captured by any tax adjustment model. Therefore there may be residual measurement error in the cigarette consumption variable, and this measurement error may increase over time due to rapid increases in real cigarette tax rates, and differential rates between states in the latter half of the sample. Prevalence of current cigarette smoking is taken from survey data which has sampling error, though this is rather small due to the large sample sizes used for the BRFSS.

The second reason is endogeneity between the dependent variable healthcare expenditure and one or more of the explanatory variables, because of two-way causation. Increased healthcare expenditure, other things being equal, may reduce the mortality rate of the population and increase the proportion of the population that is elderly though it is questionable whether the time span of the sample period is long enough for this to be a concern. There is also some recent, though mixed, evidence that per capita healthcare expenditure may affect per capita income [22,23,24,25].

If the estimated regression residuals are stationary (that is, a co-integrating regression exists) then standard methods of estimation, in this case ordinary least squares of a fixed-effects

panel model with lagged explanatory variables should produce consistent estimates even if some of the explanatory variables are endogenous or there is stationary measurement error. However, the problem of measurement error in mean cigarette consumption per smoker was considered both likely and serious enough in a finite sample with a relatively short time dimension, that we did use an instrumental variables estimator for one variable, cigarette consumption per smoker in the estimates presented in the main results. To avoid possible measurement error bias, cigarette consumption was instrumented by lagged explanatory variables. This approach was a feasible solution to the measurement error problem because cigarette stockpiling and changing market venues in response to changes in tax rates is a relatively short run phenomenon. Details of the instrumental variables estimation are presented in the Supplemental Text. Instrumental variables estimates for the all four explanatory variables that might be correlated with the regression error was done as a sensitivity analysis.

MODEL ESTIMATION

The analysis used a reduced form first order autoregression specification in which the dependent variable is expressed as a function of lagged explanatory variables, which can be used for a wide variety of stationary and nonstationary time series [4,26]. No constraints were placed on the signs of the coefficients.

The number of lags in the final model autoregression (equation 4) was determined by re-estimating it with one through four lags. Four lags, about 23 percent of average number of annual observations in the data, was considered a reasonable maximum lag order that could be precisely estimated. The first order specification with one lag was preferred by both the Akaike and Bayesian Information Criteria, two standard measures used to select the appropriate number of lags. Therefore only one lag was included in the specification of equation 1.

There is some evidence from previous research that some of the variables included in the model are nonstationary with autoregressive unit roots [1,2,3]. Other variables (particularly current smoking prevalence and mean consumption per smoker) may be stationary with high autoregressive persistence [1]. A dynamic reduced form single-equation autoregression specification [27,28] was chosen for estimation because this specification is robust to assumptions on the order of integration and the coefficients can be consistently estimated with simple one-equation techniques, such as ordinary least squares or instrumental variables. The reduced form autoregression yields unbiased coefficient estimates regardless of whether the data are stationary or nonstationary and it describes both short and long run dynamic effects [5,6,27,28,29].

The first order autoregression can be thought of as a part of a larger vector autoregression system; its main weakness is that it can produced biased predictions if some of the lagged explanatory variables are endogenous in the context of the whole system. There is no reliable formal statistical method for determining the stationarity of the time series because of the short length of the time series dimension of the sample data, so informal graphical diagnostics (time series plots and autocorrelation function and were emphasized in the determination of whether the residuals were stationary, along with one formal test for unit roots for the panel regression residuals.

The Common Correlated Effects (CCE) fixed effects estimator [4,5,6] was used to fit the panel data regression model. However, there may be three violations of the usual assumptions required for the use of ordinary least squares version of the CCE in estimating equation 4 that determined the specific CCE estimator presented in the main text and used for the sensitivity analysis. The first possible violation would be the existence of lagged error term in equation 4

which may produce endogeneity bias because the equation includes lagged explanatory variables. In particular, lagged measured mean cigarettes smoked per current smoker ($\ln(cps_{m,i,t-1})$) may be correlated with the lagged regression error term $\upsilon_{r,t} = \varepsilon_{1,i,t} + \alpha_2 \varepsilon_{2,i,t-1}$ in equation 4. (See equation 3 for source of lagged error term in $\upsilon_{r,t}$.) The second possible violation would be the existence of heteroskedasticity across states, clustered by individual state time series. This issue is handled by using robust standard errors clustered by state for the estimates. The third possible violation would be the correlation of the regression error terms across states. No estimator has been developed that solves all three problems at the same time.

After examination of the sensitivity of the coefficient and coefficient variance estimates to different assumptions required for estimating the CCE with ordinary least squares mentioned above, it was decided to that the possibility of endogeneity bias due to lags in the regression error term (equation 4 in the main text) and heteroskedasticity across states were the most important violations of standard assumptions in the variance estimation. Therefore a fixed effects panel data instrumental variables estimator with robust variance estimates clustered by states was used for the main results. Measured mean cigarette consumption per smoker $\ln(cps_{m,i,t-1})$ was instrumented by 2 and 3 period lags of itself ($\ln(cps_{m,i,t-2})$, $\ln(cps_{m,i,t-3})$), prevalence of smoking ($\ln(s_{i,t-2})$, $\ln(s_{i,t-3})$), and per capita income ($\ln(y_{i,t-2})$, $\ln(y_{i,t-3})$).

Several procedures were used to determine the suitability of the regression specification of equation 4. The Hausman test was used to test the appropriateness of the fixed versus random effects model. We checked the regression for multicollinearity and checked the residuals for cross-sectional heteroskedasticity, correlation in the regression errors across states [30], normality, and stationarity. Multicollinearity was evaluated using the Variance Inflation Factor,

and informal indications of the presence of multicollinearity, such as the sensitivity of the coefficient estimates to minor changes of specification or correlation between the explanatory variables. Scatter plots of predicted values versus the residuals were examined to determine serious regression misspecification.

An alternative CCE estimator was estimated in the sensitivity analysis to address the possibility of regression error correlation between the states; that is, that the error terms $\upsilon_{i,t}$ may be correlated across different states i . This estimator is described later in this appendix.

Statistical Calculations

Stata 12.0 [31] was used for estimation.

The main analysis for homogeneous slope pooled panel data regression was estimated the fixed effects panel data estimators using a two-stage least squares panel data instrumental variables estimator, which was implemented using the Stata add-in package xtivreg2 [32]. The xtivreg2 command includes tests for the validity of the instruments including those for weak instruments and identification. While the xtivreg2 add-in package does not correct for cross-sectional dependence in the regression errors, the evidence for the existence of dependence is ambiguous and therefore a correction may not be needed. The observed dependence may be an artifact of the small time dimension (< 20) when estimating a covariance matrix with 51 series of state regression residuals. However, the sensitivity analysis described below used an estimator that did account of possible correlation of the regression error across states did not produce statistically significant changes in the smoking behavior variables (prevalence of smoking and consumption per smoker.

The Hausman test for appropriateness of the fixed- versus random-effects estimator used the Breusch-Pagan Lagrange multiplier test, which was implemented using the post-estimation

xttest0 command in Stata [33]. Residual normality was assessed using the Stata skewness and kurtosis test for normality implemented in sktest. Cross-sectional heteroskedasticity was tested using Stata command xttest3 [34]. Cross sectional correlation in the regression error was tested with Stata command xtcsd [30] using Frees' test because the cross-sectional error terms have no intuitive pattern and contain large correlations of opposite sign. The Fisher-type inverse chi-squared test was used to test for unit roots, as implement by the Stata command xtunitroot because it can be used with panel-specific serial correlation processes, unbalanced panel data, and is most appropriate for testing in the context of the fixed, and relatively small, number of cross-sections and time series observations [35].

Only one of these three principal components for the national cross-sectional trends in the explanatory variables was significant in the panel data regression for equation 4 (main text) and that one was included in addition to the national cross-sectional average for per capital health care expenditures as variable $\ln(pc3_{ue,\, t-1})$ (Final Model, Table 1, main text). The results of the Full Model, which all cross-sectional average included in the regression, are shown in Table S2, Column A. The estimation of the Full Model showed little change from the Final Model presented in the main text.

Development of the Final Model

The Full Model (Table S2 Column A) based on equation 4 showed signs of multicollinarity, mostly involving the cross-sectional averages, severe enough to affect the precision of the estimates. To reduce multicolinearity, we performed a principal components analysis using the covariance matrix of all the cross-sectional averages included in the regression except the cross sectional average for per capita healthcare expenditures (that is, using, $s_{ue,t}$ $cps_{ue,t-1}$, $y_{ue,t-1}$, $a_{ue,t-1}$, $hs_{ue,t-1}$ $b_{ue,\, t-1}$). The cross-sectional average of healthcare expenditure (

$h_{ue,t}$) was omitted from the principal components regression because the common trends that may e correlated with it were thought to be more interpretable than the others and its behavior should be examined separately. Three principal components were significant using standard criteria (change of slope of the scree plot, percent of total variance explained, and Kaiser criterion). We then conducted a principal components regression, replacing the three principal components for the six cross-sectional averages in equation 4; the logarithm of the third component ($pc3_{ue,\, t}$) was significant at the five percent level and therefore was retained for estimation of the Final Model. An alternative approach was taken for the principal components that used the logarithmic transformation of the individual national cross-sectional variables, however this approach produced almost identical results. The regression results for the Final Regression Model (Table 1, main text) and residual diagnostics were very similar to the Full Model (Table S2, Column A). None of the differences between the elasticities of the two models differed at the five percent significance level.

ADDITIONAL DETAILED RESULTS

Model Estimates

Using the Full Model (equation 4) the estimate of the homogeneous slopes model (equation 4) is statistically significant (F(21, 50) = 195.6, P < 0.001) with good explanatory power: $R^2_{\text{within}} = 0.919$ and $R^2_{overall} = 0.509$ (Full Model, Table S2, Column A). The Hausman test rejected the appropriateness of the random effects model for both the Full and Final Models (P < 0.001), indicating that the fixed effects model is appropriate. A scatter plot of predicted per capita healthcare expenditure did not suggest regression misspecification.

In the Full Model, the elasticity of healthcare expenditure with respect to smoking prevalence, $\ln(s_{i,\,t-1})$, is 0.112 (SE 0.0319, P < 0.001) and the elasticity with respect to measured

mean cigarette consumption per smoker, $\ln(cps_{m,i,t-1})$, is 0.111 (SE 0.0271, P < 0.001) (Table S2, Column A). In the Final Model, the elasticity of healthcare expenditure with respect to smoking prevalence and measured mean cigarette consumption per smoker are 0.0118 (SE 0.0259, P < 0.001) and 0.108 (SE 0.0253, P < 0.001) (main text Table 1). The Final Model reported in the main text was used for the sensitivity analyses and the estimates of expenditure attributable to smoking behavior.

The null hypothesis of normally distributed residuals is rejected (P< 0.001 overall), though the distribution of the residuals is relatively symmetric and the rejection of normality was due to excess kurtosis (P< 0.001) which exists mostly in the interquartile range, rather than skewness (P=0.801). The null of homoscedasticity was rejected and independence of the residuals across cross-sections (across states) was also rejected at the 5% levels for all regions. The rejection of the null of cross-sectional dependence was due to sporadic large correlations with no apparent geographical or other pattern, so may be due to the small number of time observations to estimate the covariance matrix.

The null hypothesis that the residuals of all cross-sections have a unit root was rejected at the 5% level for the whole sample with and without assumption of trends in the residuals and for each region except the Far West (FW). The sensitivity analysis reported below showed that the estimated elasticities did not change substantially with the omission of the FW region and the regression residuals for the remaining BEA regions remained stationary. Therefore, the apparent non-stationarity of the residuals in the FW region did not affect results. These test results were consistent with visual examination of the residual time series plots for each state. Therefore, taking the sample as whole, the interpretation of the elastiticies as an average effect of changes in the explanatory variables across states on healthcare expenditure is reasonable.

Demographic Results

The elasticity of per capita personal income, $\ln(y_{i,t-1})$, prevalence of the population that is elderly, $\ln(a_{i,t-1})$, are 0.224 (SE 0.0674, P = 0.001), and 0.530 (SE 0.0936, P < 0.001), respectively. The elasticity of the proportion of the population Hispanic, $\ln(hs_{i,t-1})$, and proportion African-American, $\ln(b_{i,t-1})$, are 0.0108 (SE 0.00763, P =0.156), and 0.0130 (SE 0.00632, P = 0.039), respectively. The elasticity of the national trend in per capita healthcare expenditure, $\ln(h_{ue,t})$, is 0.864 (SE 0.0959, P < 0.001). No perfectly comparable specifications exist in the published literature for comparison of the estimates of the elasticity of income, $\ln(y_{i,t-1})$, and proportion of population elderly. However, studies exist with roughly similar specifications that include the coefficients for the proportion of the population elderly and variables corresponding to per capita personal income, such as per capita GDP that use similar CCE fixed effects and CCE Mean Group estimators [8,9]. The estimates for the corresponding coefficients found in this study are consistent with the lower bound of those published estimates The elasticity of the national cross-sectional average of healthcare expenditure, $\ln(h_{ue,t})$, is positive, as expected [8,9] and ranges from 0.650 to 0.864, except in the specification with flexible time trends in one of the sensitivity analysis, which attenuates the coefficient.

SENSITIVITY ANALYSES

Several sensitivity analyses, detailed in this Appendix, explored the robustness of the estimated elasticities. Equation 4 was estimated with different regression specifications (including possibility of regional variation in the elasticities), regression estimators, variance estimators, geographic sub-samples, with inclusion of flexible time trends, and inclusion of other health risk factors (including state-specific prevalence of obesity and 100 percent smoke free

laws). Other sensitivity analyses included using different instrumental variables estimators, including instrumenting the state tax variables. The results presented below are robust over these different model specifications.

None of the sensitivity analyses produced elasticity estimates for these effects of changes in prevalence of smoking and other state-specific variables that that were significantly different from the Final Model presented in the main text (P for difference between coefficients > 0.315 for all tests for current smoking prevalence and measured mean cigarette consumption per smoker; Table S2).

The estimates from the Final Model that omitted the FW region, which produced stationary residuals for all remaining regions and estimated elasticities for smoking behavior variables that are not different from the Final Model (P for difference in $\ln(s_{i,\,t-1})$ = 0.607, P for difference in $\ln(cps_{m,i,\,t-1})$ =.0.607). This result indicates that inclusion of one region with possibly non-stationary residuals did not produce unreliable estimates due to spurious correlation.

The estimator that allowed differences in all of the elasticities across BEA regions found evidence for statistically significant variation in the elasticities across BEA regions except for prevalence of current smoking which appeared to be constant across regions.

When national and regional time trends were added to the specification that allowed the elasticities to vary by region, the elasticity of measured cigarette consumption per smoker ($\ln(cps_{m,i,t})$) was 0.0679 (SE 0.0359, P = 0.059), which was also not statistically significantly different from the estimate in the Final Model (P for difference in coefficients = 0.361).

Sensitivity analysis results for the CCE estimator that do account for cross-sectional dependence, heteroskedasticity and auto-correlation in the residuals are shown in Table S2,

Column B. There were no statistically significant differences between the regression coefficients (elasticities) of the two estimators (P > 0.279 for all state specific variables, including smoking prevalence and consumption per smoker.

Attempts to instrument the state tax variables failed, either when specified as a constant elasticity across states, or allowed to vary across regions, using a variety of state specific instruments and indicators of state economic activity or population coverage by state and local 100% smokefree laws. The failure was due to the state tax variables being extremely weak instruments, which is consistent with the hypothesis that the state tax variables are exogenous over the sample period in these data. In summary, the sensitivity analysis indicated the final estimation results are robust.

Alternative Estimators

The sensitivity analysis that accounted for possible correlation between states in the regression residuals used the Stata add-in command xtscc [36,37] to estimate the Full Model (Table S2, Column B) which is robust to correlation between the regression errors for individual states in equation 4 (main text). Standard errors were estimated using robust estimates of the coefficient covariance matrix to guard against bias due to violations of the usual assumptions on regression errors, including heteroskedasticity, general serial correlation, and cross sectional correlation between cross-sectional units [36,37] (that is, individual states). The robust estimates of the coefficient covariance matrix includes lagged values of the regression residuals; the order of the lag for estimation of the robust covariance matrix may differ from the order of the lags in equation 4 (which has only one lag for the explanatory variables). Four lags of the regression residuals, $\upsilon_{r,t}$ (equation 4), were included in the robust estimate of the covariance matrix to account for possible general serial correlation in the error terms,[37] though the regression

results were not sensitive to the choice of lag length serial correlation, and possible cross-sectional correlation in the error terms [37].

A subsidiary sensitivity analysis used up to six lags of the regression residuals were included the calculation of the regression residual variance-covariance matrix. The rejection of the null hypothesis of unit roots in the residuals of all cross-sectional units was not sensitive to the number of lags included in the estimate of the residual variance-covariance matrix, therefore the choice of lags is not essential for the validity of the analysis.

Bootstrap Standard Errors

The variance-covariance matrix (and therefore standard errors) of the coefficient matrix was estimated with the bootstrap estimator. Statistical significance of the elasticities (regression coefficients) was evaluated using the bootstrap variance estimate with and without the assumption of normally distributed errors. The results assuming approximate normality are shown in Table S2, Column C. There was no change in the statistical significance of the elasticities using either assumption of normality, approximate normality, or without the assumption of normality (results not shown). The regression results in the main test are not sensitive to the non-normality of the residuals.

Inclusion of Time Trends

The effect of omitted time trends in Final Model estimate of equation 4 (main text) was explored to determine the possible effect of omitted time trends on the estimated elasticities, particular those for smoking behavior. The Final Model was first re-estimated with instrumental variables including additional terms for a national linear annual time trend, 8 regional linear annual time trends and annual national time indicator variables (to model a flexible time trend).

The residuals from the initial estimate including all time trends described above were examined for possible state-specific trends. Linear regressions were used to identify state residuals with statistically significant linear time trends using a 5% significance level. The regression was then re-estimated with thee statistically significant state-specific trends included with the other national and regional trends.

Inclusion of national, regional time trends, and state-specific time trends, did not produce any statistically or practically significant changes in the elasticities for prevalence or measured mean cigarettes smoker (Table S2, Column D) compared to the Full Model (Table 1, main text). These estimated elasticities include state linear trends following examination of the residuals from an initial estimate using BEA region-specific time trends and flexible modelling of common time trends. Therefore, these elasticties probably include the effects of overfitting of possible state trends identified in the estimated residuals, which will attribute some of the effect of smoking behavior to the existence of the hypothesized state-specific linear trends. This effect of overfitting state-specific linear trends would be that the estimated elasticities of smoking behavior for the elasticities of the state-specific variables and common trends ($\ln(s_{i,t-1})$ $\ln(cps_{i,t-1})$, $\ln(y_{i,t-1})$, $\ln(a_{i,t-1})$, $\ln(hs_{i,t-1})$, $\ln(h_{ue,t-1})$. $pc3_{i,t-1}$) would be reduced in absolute value compared to the actual elasticities due to inclusion of spurious state-specific time trends. The degree of overfitting is unknown, but this sensitivity analysis shows that the results are not sensitive to the presence of independent national, regional and state-specific time trends.

Sensitivity to Choice of Cross-Sectional Units

The Final Model was re-estimated using differently defined regions to determine stability of the estimates and effect of omission of possibly influential observations. The model was re-estimated omitting California, Arizona and Massachusetts (where longstanding and tobacco

control programs may indicate some unmeasured differences in between those states and the rest of the United States) to check whether these states were influential in the results. The model was also estimated omitting each of BEA regions in turn. Omission of Arizona, California and Massachusetts do not affect the smoking behavior elasticities in statistically or practically significant way for Final Model. The inclusion or exclusion of states with longstanding tobacco control programs, which may indicate unmeasured differences from the remaining states, and insensitivity to exclusion of individual BEA regions, made no difference in the results.

As discussed in the main text, if there is cross-sectional dependence in the residuals between states and the true coefficients (elasticities) vary across cross sectional units (that is, the states) and the residuals are nonstationary, there estimated coefficients may not represent a stable relationship between the dependent and explanatory variables. There was evidence of non-stationarity for the residuals only for states in the BEA Far West region, which was determined by conducting unit root tests on the residuals for the Far West region. Therefore in one sensitivity analysis, the Final Model was estimated without the BEA Far West region to determine whether the coefficients (elasticities) would change compared to the model including all the regions. Estimation of the Final Model excluding states in the Far West increased the coefficient for smoking behavior slightly, to 0.127 (SE 0.0260, $P < 0.001$) for smoking prevalence and to 0.137 (SE 0.0264, $P < 0.001$) for cigarette consumption per smoker. These elasticities in the estimate that omitted the FW are not statistically significantly different from those of the Final Model. The residuals for the estimate that omitted the FW estimate had time series properties similar to those for other BEA regions for the Full Model estimated including all regions (Table 1, Main Text) and appeared to be stationary. In addition, the null hypothesis of all cross-sections containing unit roots was rejected. Therefore, the apparent non-stationarity

of the residuals in the BEA Far West region did not have any practically or statistically significant effect on any of the coefficient estimates and they represent a stable relationship between the dependent and explanatory variables.

Estimation with Heterogeneous Regional Slope Coefficients

If the residuals of equation 4 are correlated across states, the regression coefficients differ across cross sectional units, and the regression residuals are not stationary, then the pooled homogeneous slope coefficients model for equation 4 may not consistently estimate the actual relationship between the explanatory variables and per capita healthcare expenditure [4]. Therefore a model that allowed the elasticities to vary by region (that is, with different, or heterogeneous, slope coefficients across regions) was estimated using a variation of the CCE fixed effects estimator, the CCE Mean Group estimator [4,5,6]. The CCE Mean Group estimator produces separate elasticity estimates for each region, which are then combined into a pooled estimate that represents an average national average effect across the states. This alternative approach to estimating the elasticities in equation 4 is more robust to assumptions about the stationarity of the residuals and variation of elasticities across regions. In particular, the presence of regional variation in the elasticities of state-specific variables in equation 4 and the stationarity of the residuals were evaluated using this alternative specification. In addition, pooled estimates showing the average national effector were compared to the model of equation 4 that assumed constant elasticities across regions.

Several methods can be used to produce pooled coefficients (elasticities) using the CCE Mean Group estimator, the most common being the simple average of the individual regional elasticities or a weighted average of regional elasticity estimates using the respective standard errors as the weights. Because of the relatively small sample size for each regional regression

and unbalanced panel data, we used a weighted average with the inverses of the variances as the weights because it was more stable and used more information from each regional estimate to obtain the pooled estimate of the national average elasticity. This method is equivalent to using standard meta-analytic methods to pool separate estimates [38], so the pooled estimates will be referred to as "meta-analytic" estimates below.

Because there are insufficient data to estimate a separate elasticity for each of the 51 states the states were grouped into the 8 BEA economic regions, and separate panel data regressions estimated using state-level data within each BEA region. The regional regression analyses yielded separate fixed effects panel data regression estimates for each of the eight regions. The regression specification was the same as equation 4, except for two changes which were necessary because the regressions were done region by region: (1) The average cigarette tax rates in the neighboring regions that were not included in the regional panel regression were included, rather than the tax rates of each individual state, and (2) individual state tax variables were included for those states that appeared in the regional panel regression. Thus, we estimated

$$\begin{aligned}\ln(h_{j,t}) &= \gamma_0 + \gamma_{0,j} + \gamma_1 \ln(s_{j,t-1}) + \gamma_2 \ln(cps_{m,j,t-1}) + \gamma_3 \ln(y_{j,t-1}) + \gamma_4 \ln(a_{j,t-1}) + \gamma_5 \ln(hs_{j,t-1}) \\ &+ \gamma_6 \ln(b_{j,t-1}) + \gamma_{tx} \ln(tx_{j,t-1}) + \gamma_7 \ln(h_{ue,t-1}) + \gamma_8 pc3_{ue,t-1} + \gamma_9 \ln(tx_{j,t-1}) + \sum_{n=1}^{N_r} \gamma_{9+n} \ln(at_{n,t-1}) + \upsilon_{i,t}\end{aligned} \tag{5}$$

where

$pc3_{ue,t}$ is the natural logarithm of the principal component of the national cross-sectional averages of the explanatory variables except per capita health care expenditure, in 2010 dollars per pack,

$\ln(at_{n,t-1})$ is the natural logarithm of the average cigarette tax in neighboring regions of *n* in year *t*, in dollars per pack,

j is individual state *i* in each region *r*,

n are the regions adjacent to region *r*, and ranges over 1,…, N_r for each region,

and other variables are defined as in the main text.

An instrumental variables estimator was used, as in the Full Model (Table S2, Column A) and Final Regression Model (Table 1, main text). The robust standard errors were calculated without clustering by states. The number of cross-sectional units (i.e., states) was too small for reliable estimation of robust standard errors with clustering by states. No systematic difference was noted between the standard errors of the robust standard errors with or without clustering by state. Besides instrumenting measured mean cigarette consumption ($\ln(cps_{m,i,t-1})$), the variable $\ln(tx_{j,t-1})$ was instrumented and the lagged values included as excluded instruments ($\ln(tx_{j,t-2})$, $\ln(tx_{j,t-3})$). The rationale for the choice of instrumented variables and instruments was the same as in the main text

Note that the regional tax variables $\ln(at_{n,t-1})$ differ from those in the homogeneous elasticity models because all 51 states could not be included in separate regional regressions, therefore additional variables were added to the regression for the average regional cigarette tax rates for states outside of the region.

The meta-analytic estimates consist of eight separate regressions for the eight BEA regions, rather than the whole sample. The regression coefficients for each variable from each regional panel regressions were pooled using the inverse of the variance of the coefficient estimates was weights used to tested the null hypothesis of homogeneity using the chi-square test [38]. If homogeneity was rejected at the 5% significance level a random effects pooled estimate was calculated using the DerSimonian-Laird method [39]. Note that the term “random effects” in the context of pooling effect sizes refers to any excess variation between estimated statistics that cannot be explained by random sampling variation and differs from the distinction between random and fixed effect estimators in the context of panel data regression.

The same residual diagnostics were performed as for the pooled homogenous slope coefficient model described in the main text. Sensitivity analysis on inclusion of cross-sectional units, and inclusion of obesity were conducted.

The heterogeneous elasticities calculated from separate regional panel regressions, each using individual state data, produced statistically significant elasticities for several state-specific variables and the common trend for national average per capita healthcare expenditures. The null of homogeneity across regions was rejected for all coefficients at the 5% significance level, except for cigarette prevalence, $\ln(s_{j,t-1})$, state tax level, $\ln(tx_{j,t-1})$, and national cross-sectional average of per capita healthcare $(h_{ue,t-1})$. The pooled elasticities, computed as random or fixed effects as indicated by the homogeneity test, for current smoking prevalence, $\ln(s_{s,t-1})$, mean per capita cigarette consumption, $\ln(cps_{m,s,t-1})$, per capita income, $\ln(y_{s,t-1})$, proportion of the population elderly, $\ln(\alpha_{s,t-1})$, and cross-sectional average healthcare expenditure, $(h_{ue,t-1})$, are 0.144 (SE 0.0193, P < 0.001), 0.0893(SE 0.0311, P =0.004), 0.238 (SE 0.0475, P < 0.001), 0.438 (SE 0.0104, P < 0.001, and 0.646 (SE 0.0522, P < 0.001), respectively (Table S2, column E). The elasticities for the smoking behavior variables are not statistically significantly different from the Final Model (Table 1, main text), as discussed in the main text. The coefficient estimates are similar to those for the panel regression that assumed homogenous coefficients across regions, except it was possible to estimate a statistically significant pooled state-specific (rather than regional) cigarette tax coefficient.

The residuals for each regional panel regression appeared to be more stationary compared the Full Model (Table S2) and Final Regression Model (Table 1, main text) when examined region by region; this was true for both visual and graphical examination and by panel unit root

tests. The overall R^2 for the fitted values of meta-analytic estimator is 0.641, considerably higher than for the homogenous slopes models (0.495 for Final Model, Table 1 in main text, 0.509 for the Full Model, Table S2, Column B). Residuals for some, but not all, regions displayed heteroskedasticity and dependence of the regression residuals across states. The null hypothesis that each regional regression panel had a unit root was rejected for all regions except the Far West region. Re-estimation without the Far West region produced a coefficient of 0.148 (SE 0.0199, P <0.001) for prevalence of smoking, and 0.106 (SE 0.0313, P < 0.001) for cigarette consumption per smoker.

These results show that there is evidence that some elasticities vary across states, and the regression residuals are stationary. The pooled elasticities for smoking prevalence and measured mean cigarette consumption per smoker with and without the Far West Region are not statistically different from the Final Model (Table 1, main text). There is no statistically or practically significant difference between the estimated elasticities describing average national behavior under the assumption of constant or varying elasticities across regions. There is evidence for non-stationarity in only one region (the Far West) and omission of that region makes no practically or statistically significant difference in the estimated elasticities of the smoking behavior variables. Therefore this sensitivity analysis supports the conclusion that there is a stable relationship between per capita healthcare expenditures and the smoking behavior variables: current smoking prevalence and cigarette consumption per smoker.

Instrumental Variables Estimator for Measurement Error in Prevalence of Current Smoking

The prevalence of current smoking, $s_{i,t}$, is taken from BRFSS survey results and has a published sample standard error that varies slightly from state to state. The relative standard error

(standard error / mean) in the measured prevalence over the sample period was small, averaging 4.3% and ranging from a minimum of 1.8% to a maximum of 8.5%. The presence of sample error in the estimate of prevalence of current smoking creates an "errors in variables problem" that could bias the estimated elasticity of current smoking, even in a within fixed effects estimator. Analysis of the possible bias is difficult with panel data that are non-stationary or trending. Generally, the estimated elasticity of current smoking prevalence with measurement and perhaps high autocorrelation in the data would be expected to produce estimates that are biased downward towards zero. Therefore, the estimated elasticity of a variable with measurement error will underestimate the true elasticity [40,41]. To evaluate this possible bias we estimated the Final Model (Table 1, main text) with prevalence of current smoking instrumented in addition to cigarette consumption per smoker. Any bias of the estimated elasticity will decrease as the autocorrelation coefficient between the instrumented variable $(s_{i,t-1})$ and its lagged instruments $(s_{i,t-j})$ decreases as j increases.

Estimates were calculated with $s_{i,t-2}$, $s_{i,t-3}$, and $s_{i,t-4}$ as instruments for $s_{i,t-1}$. The estimated elasticity for prevalence of current smoking did increase as the length of the difference in lag between the instrumented variable $(s_{i,t-1})$ and the lagged instruments increased, which was expected. The estimated elasticity for prevalence of current smoking ranged from 0.177 (SE 0.0818, P = 0.030) to 0.347 (SE 0.229, P = 0.129). These estimates did not differ from those of the Final Model (Table 1, main text) at the 5% significance level. They do indicate that the measurement error for the prevalence of current smoking may have biased the estimated coefficient in the Final Model toward zero somewhat, so that the estimated effect of changes in smoking prevalence on healthcare expenditure may underestimate the true effect. The extent of this bias is difficult to evaluate statistically. Intuitively, severe downward bias towards zero with

the within fixed effects estimator would occur when measurement error is proportionally a large component of the observed variation in a variable observed with measurement error that dominates the real movements of the variable [41]. It is unlikely that the observed downward trends and autocorrelated variation in prevalence in individual states around that downward trend are mostly sampling error over sample periods of over ten or more years.

Instrumental Variables Estimation

The estimates are not sensitive to the presence of endogeneity in the explanatory variables or stationary measurement errors. The standard criteria for selection of lag order for the whole country (all of the states) indicates one lag is appropriate. Therefore, the best autoregression that approximates the true model for the whole country has one lag in the explanatory variables. There is good evidence that the explanatory variables are non-stationary or trending in a way that will make them act non-stationary in a relatively short time series, so we must estimate a cointegrating vector to achieve stationary regression errors.

The coefficients of individual terms in an autoregression are short run effects of each variable. The long run effects of a given variable are just the sums of the short run coefficients of that variable across the lags. In an autoregression that contains a cointegrating vector with one lag, the coefficients of the lagged explanatory variables will be same as those in the long run relationship described by the cointegrating vector. The short run dynamics (over a period of one year) will involve only the dependent variable (in this case, per capita healthcare expenditure). Therefore, for a parsimonious model that is a good approximation for the whole country (all of the states) the estimated coefficients for the explanatory variables are the long run cointegrating vector coefficients. Since they are long run cointegrating vector coefficients, the coefficient estimates will be insensitive to choice of instruments in the presence of endogeneity or stationary

measurement error. Furthermore, using lagged explanatory variables reduced the problem of endogeneity and measurement error in a finite (though large) sample, because a correlation of the explanatory variables and the regression error term would occur with past autocorrelated tersms of the regression error, not the regression error term itself.

We chose to instrument per capita cigarette consumption because we thought the widespread trend rise in real state cigarette tax rates after 2000 and in real differences in tax rates between different individual states would lead to non-stationary measurement error. Because of changing BRFSS sample design and trends in standard errors of the survey estimates, the sensitivity analysis using instruments for prevalence of cigarette smoking was also conducted for the reasons explained in the main text.

In the sensitivity analysis that included all of the variables that may be correlated with the regression error, per capita income and the proportion of population 65 years or older (for which there is strong evidence of non-stationarity) were instrumented with artificially generated irrelevant instrumental variables that, by construction, cannot be correlated with a stationary regression error term [42].

First Stage Estimates and Instrumental Variables Diagnostics for Final Regression Results in the Main Text (Table 1)

For the final regression model results in the main text (Table 1), the first stage estimates are the estimates of cigarette consumption per smoker using the instruments, adjusted for the other explanatory variables (Table S3). The first stage estimates are used to evaluate whether the instruments have sufficient power to explain cigarette consumption per smoker and whether they are properly excluded from the autoregression. Following the first stage regression estimates are the second stage estimates, which are the same as those presented in the main text, Table 1. The

second stage, in effect, uses the predicted value of cigarette consumption per smoker (predicted using the instruments), as an instrument for the observed value of cigarette consumption per smoker. Table S3 presents the detailed second stage results and the diagnostic tests for the instruments.

The model estimates presented in the main text are identified and the instruments are not weak, however, they do not pass formal over-identification tests in either the first or second stage (in Stata, the Hansen J statistic) estimates (the joint null hypothesis that the instruments are correctly excluded from the model and are valid instruments is rejected with p = 0.0165). These results reject the joint null that the model is at least exactly identified and the instruments are valid. This rejection is probably due to the failure of the assumption that the instruments (which are additional lags of the explanatory variables) are correctly omitted from the model for a few of the states. In other words, there are a few states were, when looked at separately, where lags of order 2 should be included in the model, even though for all the states in the whole sample, a one lag is indicated by conventional model selection criteria, and one lag of the explanatory variables is the best approximation to the true model for the whole country. Therefore, the results of the over-identification test may be misleading. A sensitivity analysis is required to determine the sensitivity of the results to the choice of instruments. The results of the sensitivity analysis indicate that the results presented in the main text are not sensitive to the failure of the over-identification test in this case.

Instrumental Variables Estimator Sensitivity Analysis

In the results in the main text, we used instrumental variables estimation only for cigarette consumption per current smoker because of large increases in real cigarette tax rates and growing discrepancies in tax rates between states and regions towards the end of the sample.

Those large increases suggested that measurement error for cigarette consumption per smoker (that is, untaxed consumption) might be increasing towards the end of the sample and act as if it were nonstationary.

Our main concern was stationarity of the residuals. Prior research [1,2,3] indicated that the variables included in the analysis are nonstationary or trending in a way that makes them act as if they were non-stationary in relatively short time series. Standard information criteria indicated that the appropriate order of the autoregression as a model for the whole sample (that is, all the states) is one lag. Therefore the behavior of the autoregression is dominated by a cointegrating vector that models the long run relationship, with short run dynamics involving per capita healthcare. Therefore, the estimated regression coefficients should be insensitive to issues of endogeneity and instrumentation, and stationary measurement error. Moreover, with lagged state-specific explanatory variables endogeneity should be a problem only through serial correlation in the error term even with stationary variables.

We did three sensitivity analyses to determine the sensitivity of the results to endogeneity bias and measurement error. The first sensitivity analysis used the property that a panel autoregression (a regression that used lagged explanatory variables) will be sensitive to endogeneity bias and measurement error through autocorrelation in the panel regression error term. We examined the sensitivity of the coefficients to the autocorrelation in the panel data regression residual, and then conducted a specification search to improve the performance of the overidentification test diagnostic for the instruments. The second sensitivity analysis used irrelevant instrumental variables estimates that eliminated the usual sources of bias induced by invalid instruments. The third sensitivity analysis estimated the cointegrating regression representing the long-run relationship, which is insensitive to choice of instruments, with

ordinary least squares. These sensitivity analyses produced coefficient estimates that are consistent with the interval estimates shown in the Final Regression Model shown in Table 1 in the main text.

The first analysis explored the possible endogeneity bias caused by correlation between the lagged explanatory variables and serial correlation in the regression errors. We divided the regression residuals into thirds -- low mid and high serial correlation -- and estimated the specification in the main text for each third. We saw no pattern of bias in the estimated coefficients as a function of the degree of serial correlation in the regression residuals [29]. The models were identified and instruments for the cigarette consumption per smoker were not weak for any of these regressions. The estimates passed the over-identification test for the low- and mid-serial correlation states, but not for the high correlation states. Selected results are shown in Table S4.

We suspected that the rejection of the null for the over-identification test was the presence of longer lags in the true autoregression among the one third of states with high serial correlations in the residuals. Standard information criteria indicated one lag was appropriate for the country as a whole. However, these same criteria indicated that a second lag was present for the states with high residual serial correlation in these states, specifically real per capita personal income and percentage of population that is African-American. A short specification search produced the regression shown in Table S5.

The results of the specification search in Table S5 show that the regression equation is identified, the instruments are not weak, and the null of the overidentification tests is accepted, indicating that the equation is overidentified and the instruments are valid. The first stage regression has an R^2 of 0.78 with an F-test $p< 0.0001$, the Kleibergen-Papp rk LM statistic has

p< 0.0001, and the corresponding F-statistic for weak identification is 43.98. The first stage over-identification test is strongly rejected (p< 0.001) which suggests that after the specification search, there is some evidence that some of the instruments should be included in the autoregression. A problem is correctly identifying the order of the lags for all the states which may differ between states (that is different second or higher lags may exist for some states). In addition, the first stage regression is more sensitive to that mispecification. However, the coefficients for the smoking variables are close to those presented in the main text. The p-value for the difference between the coefficients in the Table S5 regressions and those in the main text are 0.434 for cigarette consumption per smoker, and 0.540 for prevalence of smoking.

The results for the second sensitivity analysis re-estimating the Final Regression Results presented in Table 1 with prevalence of smoking and cigarette consumption per smoker instrumented with irrelevant instrumental variables are shown in Table S6. As far as we know, there is no theory of efficient estimation in fixed effects panels for irrelevant instruments, so these estimates may be quite inefficient. There is no correlation due to structural relationships between the data and the instruments because the instruments were calculated from arbitrary basis functions. Therefore we are interested in comparing the point estimates to the confidence intervals of the final regression results in Table 1 in the main text. The estimated coefficient using irrelevant instruments for the prevalence of smoking is 0.170 and is very close to the upper confidence limit for the corresponding coefficient of the final regression results in Table 1. The estimated coefficient for cigarette consumption per smoker from the irrelevant instruments estimate is 0.0849, which is far within the 95% confidence interval for the corresponding coefficient in final regression results in Table 1.

The third sensitivity analysis uses an ordinary least squares estimate of the cointegrating regression similar to the final regression model reported in Table 1 but with with unlagged explanatory variables provides similar estimates as those in Table 1. The estimated coefficient for prevalence of smoking in the cointegrating regression is 0.107 (SE 0.0348) and for cigarette consumption per smoker is 0.0823 (SE 0.0241). These values are close to the corresponding values in Table 1 of 0.118 (SE 0.0259) and 0.108 (SE 0.0253). The coefficients for the other variables are also similar to those in Table 1. This result is precisely what one would expect from a cointegrating regression with a large number of observations.

It is very difficult, indeed, rarely possible, to definitively answer questions about the validity of instruments used in instrumental variables regressions using formal statistical tests using in-sample statistical tests [43]. The reason for this uncertainty is that the reliability of tests on instruments, particularly over-identification tests is dependent on joint hypotheses, parts of which must remain an untested part of the joint hypotheses for the tests to be informative on the questions of most interest, in this case whether the instruments are uncorrelated with the regression error term. This issue is particularly difficult when the maintained hypothesis involved the correct order of an autoregressive specification in a panel data analysis, where some aspects of the model specification must be an approximation. Often, questions involving the quality of instrumental variables estimates must rest on the stability of the results of several sensitivity analyses and the plausibility of the initial choice of instruments.

Effect of Weighting Scheme on Regional Healthcare Expenditures Attributable to Smoking

The data and statistical analysis in Table 2 in the main text used an approach to aggregation from state to regional elasticity estimates that is consistent with the units of observation used in the regression analysis, which implied that each state be given equal weight,

and estimates of burden be calculated using equal weights rather than being weighted by population. This equal weighting procedure is appropriate for the tobacco control policy choices a state or regional level policy maker faces in decision making using the evidence from the results of 51 state experiments with varying the levels of current smoking prevalence and mean cigarette consumption per smoker in a way that would not be distorted by a few states with a large populations.

Another approach would be to use population weighted estimates of the effects of differences in smoking on differences in healthcare expenditure. These population-weighted estimates are shown in Table S7. The estimates in Table S7 are similar to those in Table 2 in the main text, with the principal exception of New England, where the estimates for a single state, Massachusetts, dominate the estimates for the whole region because of its large population relative to the rest of New England. The estimates of excess burden for New England may be too high in the population weighted estimates (Table S7) if they are dominated by one state, Massachusetts. Modelling the possible state-specific effects of unmeasured consumption due to tax differentials was not possible due to severe multicollinearity in the explanatory variables for New England, especially Massachusetts. The population-weighted estimates were similar to those of the equal weighted estimated, except that states with large populations relative to the regional population had a larger effect on the results.

STATE-SPECIFIC HEALTHCARE EXPENDITURES ATTRIBUTABLE TO SMOKING

The state-specific estimates of excess burden are shown in Table S8, calculated as described in the main text. These results should be considered cautiously because the individual estimates of the effect of state cigarette tax differentials and proportion of measured cigarette consumption per smoker that is due to estimated unmeasured state consumption may be

imprecise for some states for the following reasons. First, these estimates of excess burden apply estimates of the national average elasticities of state level measures of smoking behavior on per capita healthcare expenditure variables to individual states and the national average regression model may not model each specific state with equal accuracy. There is evidence of regional differences in elasticities from the sensitivity analysis, however, which model is most accurate for modeling state specific behavior cannot be determined from formal in-sample statistical tests and requires an analysis of out-of-sample predictions for individual states [40]. Detailed modeling of the effects of state tax differentials for individual states was not possible with the available data due to severe multicollinearity problems among state-specific variables and the relatively short number of annual observations for individual states and small groups of states. Second, separate estimates for 51 individual cross-sectional units are likely to include some inaccurate in-sample predictions due to random variation. Third, some of the estimated cigarette tax elasticities are imprecise due to limitations in the available state-level data. This imprecision is not important for estimates for many states, but when the combined effect of differences in prevalence of smoking and consumption per smoker from the national average is close to zero for a state, this imprecision in estimation of the tax effect may have a large impact on the estimates of total excess burden.

COMPARISON TO PREVIOUS RESULTS

As noted in the Discussion section in the main text, this research is not directly comparable to previous studies done on California [1,2] and Arizona [3]. The main reason is that the previous research uses a large group of 38 different control states that were compared rather than the cross-sectional averages of all states for statistical adjustment. This previous research also did not use per capita expenditure of the resident population as the measure of healthcare

expenditure. However, to the extent that the observable and correlated unobservable common trends in the 39 control states reflect the common national trends that are at the core of the model presented in this paper, a comparison of these results to previous results may be of interest.

Depending on the measure of healthcare expenditure used, the elasticity of per capita healthcare expenditure with respect to the prevalence of current smoking in previous research in California [1] is between 0.105 (SE 0.0293) (P = 0.740 for difference from corresponding estimate in Final Regression Results, Table 1) and 0.200 (SE 0.217) (P = 0.707 for difference from corresponding estimate in Final Regression Results); the elasticity with respect to mean cigarette consumption per current smoker is between 0.152 (SE 0.0381) (P = 0.460 for difference from corresponding estimate in Final Regression Results) and 0.271 (SE 0.0859) (P = 0.088 for difference from corresponding estimate in final regression results) The point estimates of the elasticities in California may be somewhat higher than in the rest of the United States, though not statistically significantly different at the 5% level).

VARIATION IN PREVALENCE OF SMOKING AND CONSUMPTION PER SMOKER

Estimates of the effect of changes up to 10% are given for changes in both prevalence of current smoking $(s_{i,t})$ and cigarette consumption per smoker $(cps_{i,t})$ are given in the main text. Evidence is presented below that the final regression results estimates (Table 1, main text) are valid for changes of up to 10% because these changes are well within the range of variation observed in the sample for 1992 to 2009 using two alternative measures. The first is the absolute value of the proportional variation of each variable around each state's mean, which is the relevant measure of variation for the within fixed-effects CCE estimator. This measure of variation may be questioned, since the data are strongly trending and some variables appear to be non-stationary. Therefore a second measure is presented, the absolute value of the proportional

annual change in each variable, where for example, the annual proportional change in the prevalence of current smoking is calculated as

$$\left|\frac{(s_{i,t}-s_{i,t-1})}{((s_{i,t}+s_{i,t-1})/2)}\right|.$$

A ten percent change is within the range of variation of the data over the sample period for both prevalence of smoking and cigarette consumption per smoker for both measures. For prevalence of current smoking, a 10% change in prevalence is at approximately the 75th percentile of the absolute value proportional variation around each state mean, and approximately the median for consumption per current smoker. For the absolute values of the proportional annual change, 10% is at approximately the 90th percentile for prevalence of current smoking, and approximately the 75th percentile for cigarette consumption per current smoker (Table S9).

REFERENCES

1. Lightwood J, Glantz S (2013) The Effect of the California Tobacco Control Program on Smoking Prevalence, Cigarette Consumption, and Healthcare Costs: 1985-2008. PLoS ONE 8: e47145.
2. Lightwood JM, Dinno A, Glantz SA (2008) Effect of the California Tobacco Control Program on Personal Health Care Expenditures. PLoS Medicine 5: e178.
3. Lightwood J, Glantz S (2011) Effect of the Arizona Tobacco Control Program on Cigarette Consumption and Healthcare Expenditures. Social Science and Medicine 72: 166-172.
4. Ebarhardt M (2009) Nonstationary Panel Econometrics and Common Factor Models: An Introductory Reader. Oxford, UK: Department of Economics, Oxford University. 1-60 p.
5. Kapetanios G, Pesaran M, Yamagata T (2009) Panels with Nonstationary Multifactor Error Structures. Cambridge, UK: Department of Ecnomics, University of Cambridge.
6. Pesaran M (2006) Estimation and Inference in Large Heterogeneous Panels with a Multifactor Error Structure. Econometrica 74: 967-1012.
7. Warner KE, Hodgson TA, Carroll CE (1999) Medical Costs of Smoking in the United States: Estimates, Their Validity, and Their Implications. Tobacco Control 8: 290-300.
8. Baltagi B, Moscone F (2010) Health Care Expenditure and Income in the OECD Reconsidered: Evidence from Panel Data. Economic Modelling 27: 804-811.
9. Moscone F, Tosetti E (2010) Health Expenditure and Income in the United States. Health Economics 19: 1385-1403.
10. Bethel JW, Schenker MB (2005) Acculturation and Smoking Patterns among Hispanics: A Review. Am J Prev Med 29: 143-148.

11. Mayberry RM, Mili F, Ofili E (2000) Racial and Ethnic Differences in Access to Medical Care. Med Care Res Rev 57 Suppl 1: 108-145.

12. Becares L, Shaw R, Nazroo J, Stafford M, Albor C, et al. (2012) Ethnic Density Effects on Physical Morbidity, Mortality, and Health Behaviors: A Systematic Review of the Literature. Am J Public Health 102: e33-66.

13. Dubay LC, Lebrun LA (2012) Health, Behavior, and Health Care Disparities: Disentangling the Effects of Income and Race in the United States. International Journal of Health Services 42: 607-625.

14. Ai AL, Noël LT, Appel HB, Huang B, Hefley WE (2013) Overall Health and Health Care Utilization among Latino American Men in the United States. Am J Mens Health 7: 6-17.

15. Lightwood J, Glantz S (2011) Predicted Effect of California Tobacco Control Funding on Smoking Prevalence, Cigarette Consumption, and Healthcare Costs, 2012-2016. San Francisco, CA: Center for Tobacco Control Research and Education, University of California, San Francisco.

16. Breitung J, Pesaran M (2008) Unit Roots and Cointegration in Panels. In: Matyas L, Sevestre P, editors. The Econometrics of Panel Data. Berlin: Springer-Verlag. pp. 279-322.

17. Orzechowski W, Walker RC (2007) The Tax Burden on Tobacco-Historical Compilation, Vol. 42. Arlington, VA: Orzechowski & Walker.

18. Orzechowski W, Walker RC (2009) The Tax Burden on Tobacco, Vol. 44. Arlington, VA: Orzechowski & Walker.

19. Centers for Disease Control and Prevention (2009) Federal and State Cigarette Excise Taxes - United States, 1995-2009. MMWR Morb Mortal Wkly Rep 58: 524-527.

20. Centers for Disease Control and Prevention (CDC). State Tobacco Activities Tracking and Evaluation (State) System. Centers for Disease Control and Prevention http://apps.nccd.cdc.gov/statesystem/Default/Default.aspx. (Accessed Feb 18)

21. Bureau of Economic Analysis. Regional Economic Accounts: Methodologies <Url: Http://Www.Bea.Gov/Regional/Methods.Cfm>. Bureau of Economic Analysis http://www.bea.gov/regional/methods.cfm. (Accessed

22. Amiri A, Ventelou B (2012) Granger Causality between Total Expenditure on Health and Gdp in Oecd: Evidence from the Toda-Yamamoto Approach. Economics Letters 116: 541-544.

23. Devlin N, Hansen P (2001) Health Care Spending and Economic Output: Granger Causality. Applied Economics Letters 8: 561-564.

24. Erdil E, Yetkiner IH (2009) The Granger-Causality between Health Care Expenditure and Output: A Panel Data Approach. Applied Economics 41: 511-518.

25. French D (2012) Causation between Health and Income: A Need to Panic. Empirical Economics 42: 583-601.

26. Maddala GS, Kim I-M (1998) Unit Roots, Cointegration, and Structural Change. Cambridge: Cambridge University Press.

27. Hsiao C (2001) Identification and Dichotimization of Long- and Short-Run Relations of Cointegrated Vector Autoregressive Models. Econometric Theory 17: 889-912.

28. Hsiao C (2006) Cowles Commission Structural Equation Approach in Light of Nonstationary Time Series Analysis. In: No H-C, Ing C-K, Lai TL, editors. Time Series and Related Topics: In Memory of Ching-Zong Wei. Beachwood, Ohio: Institute of Mathematical Statistics. pp. 173-192.

29. Enders W (2004) Applied Econometric Time Series. Hoboken, NJ: John Wiley and Sons.

30. De Hoyos RE, Sarafidis V (2006) Testing for Cross-Sectional Dependence in Panel-Data Models. The Stata Journal 6: 482–496.

31. StataCorp LP (2011) Stata Version 12. College Station, Texas.

32. Schaffer ME. Xtivreg2: Stata Module to Perform Extended Iv/2sls, Gmm and Ac/Hac, Liml and K-Class Regression for Panel Data Models <Url: Http://Ideas.Repec.Org/C/Boc/Bocode/S456501.Html>. (Accessed

33. Breusch T, Pagan A (1980) The Lagrange Multiplier Test and Its Applications to Model Specification in Econometrics. Review of Economic Studies 44: 239-253.

34. Baum CF (2001) Residual Diagnostics for Cross-Section Time Series Regression Models. The Stata Journal 1: 101–104.

35. StataCorp LP (2011) Stata Longitudinal Data / Panel Data Reference Manual, Release 12. College Station, Texas: Stata Press.

36. Driscoll J, Kraay A (1998) Consistent Covariance Matrix Estimation with Spatially Dependent Panel Data. The Review of Economics and Statistics 80: 549-560.

37. Hoechle D (2007) Robust Standard Errors for Panel Regressions with Cross-Sectional Dependence. The Stata Journal 7: 281-312.

38. Shadish WR, Haddock WR (1994) Combining Estimates of Effect Size. In: Cooper H, Hedges LV, editors. The Handbook of Research Synthesis. New York, NY: Russell Sage Foundation. pp. 261-281.

39. DerSimonian R, Laird N (1986) Meta-Analysis in Clinical Trials. Controlled Clinical Trials 7.

40. Hsiao C (2003) The Analysis of Panel Data, 2nd Edition. Cambridge, UK: Cambridge University Press.

41. Johnston J, DiNardo J (1997) Econometric Methods, 4th Ed. New York, NY: McGraw-Hill.

42. Phillips PCB (2006) Optimal Estimation of Cointegrated Systems with Irrelevant Instruments. New Haven, CT: Cowles Foundation, Yale University.

43. Murray M (2006) Avoiding Invalid Instruments and Coping with Weak Instruments. Journal of Economics Perspectives 20: 111-132.

Table S1. States in Bureau of Economic Analysis (BEA) Economic Regions	
New England Region (NE)	Connecticut, Maine, Massachusetts, New Hampshire, Rhode Island, Vermont
Mideast Region (ME)	Delaware, District of Columbia, Maryland, New Jersey, New York, Pennsylvania
Great Lakes Region (GL)	Illinois, Indiana, Michigan, Ohio, Wisconsin
Plains Region (PL)	Iowa, Kansas, Minnesota, Missouri, Nebraska, North Dakota, South Dakota
Southeast Region (SE)	Alabama, Arkansas, Florida, Georgia, Kentucky, Louisiana, Mississippi, North Carolina, South Carolina, Tennessee, Virginia, West Virginia
Southwest Region (SW)	Arizona, New Mexico, Oklahoma, Texas
Rocky Mountain Region (RM)	Colorado, Idaho, Montana, Utah, Wyoming
Far West Region (FW)	Alaska, California, Hawaii, Nevada, Oregon, Washington

Table S2. Sensitivity Analysis Results, CMS state resident healthcare expenditure, 1992-2009					
	A	B	C	D	E
Specification	Full Specification	Full Specification	Full Specification	Principal Components with regional, state and flexible time trends	Meta-analytic estimates based on BEA regional panel regressions
Variance Estimation	Clustered by states, robust to cross-sectional heteroskedasticity (Full Model)	Cross-sectional dependence and general autorcorrelation processes	Bootstrap standard errors	Clustered by states, robust to cross-sectional heteroskedasticity	Clustered by states, robust to cross-sectional heteroskedasticity
Estimator	IV	OLS with robust standard errors	IV	IV	IV
R^2					
within	0.918	0.910	0.918	0.958	--
between	0.290	0.307	0.290	0.194	--
overall	0.509	0.505	0.509	0.470	0.624
ρ	0.942	0.941	0.942	0.970	--
corr(ui,Xb)	-0.341	-0.366	-0.341	-0.261	--
$\ln(s_{i,\,t\text{-}1})$	0.112	0.0859	0.112	0.0879	0.144
SE	(0.0319)	(0.0303)	(0.0382)	(0.0197)	(0.0193)
p	< 0.001	0.007	0.003	< 0.001	< 0.001
$\ln(cps_{m,\,i,\,t\text{-}1})$	0.111	0.0710	0.111	0.0988	0.0893
SE	(0.0271)	(0.0268)	(0.0355)	(0.0136)	(0.0311)
p	< 0.001	0.011	0.002	< 0.001	0.004
$\ln(y_{i,\,t\text{-}1})$	0.306	0.316	0.306	0.265	0.238
SE	(0.112)	(0.0518)	(0.114)	(0.0634)	(0.0475)
p	0.006	< 0.001	0.007	< 0.001	< 0.001
$\ln(a_{i,\,t\text{-}1})$	0.568	0.597	0.568	0.446	0.438
SE	(0.0870)	(0.0380)	(0.118)	(0.0626)	(0.0104)
p	< 0.001	< 0.001	< 0.001	< 0.001	< 0.001
$\ln(hs_{i,\,t\text{-}1})$	0.0132	0.0101	0.0132	0.00526	0.0107
SE	(0.00851)	(0.00368)	(0.00876)	(0.00443)	(0.00880)
p	0.121	0.008	0.132	0.235	0.222
$\ln(b_{i,\,t\text{-}1})$	0.0146	0.0134	0.0146	0.00798	0.00608
SE	(0.00630)	(0.00443)	(0.00657)	(0.00409)	(0.00920)
p	0.020	0.004	0.026	0.051	0.511
$\ln(tx_{i\,,\,t\text{-}1})$	--	--	--	--	0.0394
SE	--	--	--	--	(0.0114)
p	--	--	--	--	0.001
$\ln(tx_{i\,\varepsilon NE,\,t\text{-}1})$	0.0461	0.0424	0.0461	0.0222	-0.0851
SE	(0.0103)	(0.00527)	(0.0126)	(0.00825)	(0.0619)
p	< 0.001	< 0.001	< 0.001	0.007	0.169
$\ln(tx_{i\,\varepsilon ME,\,t\text{-}1})$	0.0166	0.00753	0.0166	0.0295	0.0230
SE	(0.0101)	(0.00683)	(0.0168)	(0.0123)	(0.0304)
p	0.102	0.276	0.323	0.016	0.434

Table S2. Sensitivity Analysis Results, CMS state resident healthcare expenditure, 1992-2009 (Continued)					
	A	B	C	D	E
Specification	Full Model	Full Specification	Full Specification	Principal Components with regional, state and flexible time trends	Pooled Meta-analytic estimates based on BEA regional panel regressions
Variance Estimation	Clustered by states, robust to cross-sectional heteroskedasticity	Cross-sectional dependence and general autorcorrelation processes	Bootstrap standard errors	Clustered by states, robust to cross-sctional heteroskedasticity	Not clustered by states, robust to cross-sectional heteroskedasticity
Estimator	IV	OLS	IV	IV	IV
$\ln(tx_{i\,\varepsilon\,GL,\,t-1})$	-0.00623	-0.0129	-0.00623	0.00803	0.0487
se	(0.0163)	(0.00843)	(0.0217)	(0.0089)	(0.0210)
p-value	0.702	0.132	0.774	0.367	0.020
$\ln(tx_{i\,\varepsilon\,PL,\,t-1})$	0.0275	0.0212	0.0275	0.0062	-0.0484
se	(0.0174)	(0.0216)	(0.0248)	(0.0127)	(0.137)
p-value	0.114	0.332	0.267	0.625	0.723
$\ln(tx_{i\,\varepsilon\,SE,\,t-1})$	0.00894	0.00961	0.00894	-0.0364	0.0831
se	(0.0236)	(0.0101)	(0.0248)	(0.0148)	(0.0700)
p-value	0.705	0.348	0.719	0.014	0.235
$\ln(tx_{i\,\varepsilon\,SW,\,t-1})$	-0.00558	-0.0173	-0.00558	0.0340	0.0253
se	(0.0237)	(0.0162)	(0.0282)	(0.0119)	(0.0133)
p-value	0.814	0.291	0.843	0.004	0.056
$\ln(tx_{i\,\varepsilon\,RM,\,t-1})$	-0.0216	-0.0306	-0.0216	-0.00252	0.0283
se	(0.0317)	(0.00622)	(0.0434)	(0.0218)	(0.0295)
p-value	0.171	< 0.001	0.259	0.796	0.295
$\ln(tx_{i\,\varepsilon\,FW,\,t-1})$	0.0164	0.00415	0.0164	0.0153	-0.0171
se	(0.0317)	(0.0134)	(0.0434)	(0.0218)	(0.186)
p-value	0.606	0.757	0.706	0.481	0.927
$\ln(h_{ue,\,t-1})$	0.727	0.732	0.727	-0.290	0.650
se	(0.0659)	(0.0492)	(0.0666)	(0.403)	(0.0851)
p-value	< 0.001	< 0.001	< 0.001	0.472	< 0.001
$pc3_{ue,\,t-1}$	--	--	--	-0.136	-0.380
se	--	--	--	(0.515)	(0.216)
p-value	--	--	--	0.792	0.080
Constant	--	0.932	1.00	--	-0.378
se	--	(0.487)	(0.523)	--	0.217
p-value	--	0.062	0.055	--	0.080

Standard errors are in parentheses.
Coefficients are interpreted as elasticities.
ρ: proportion of regression error variance due to cross-sectional state-specific constants.
Corr (u_i, Xb): Correlation between linear state-specific intercept and linear score.
Dependent Variable: natural log of per capita healthcare expenditures in 2010 $.

Table S3. First and second stage estimates and instrumental variables diagnostics for Final Regression Results (Table 1) in main text

```
FIXED EFFECTS ESTIMATION
------------------------
Number of groups =        51                    Obs per group: min =        13
                                                               avg =      17.5
                                                               max =        18

First-stage regressions-----------------------
First-stage regression of llcps_s:
FIXED EFFECTS ESTIMATION
------------------------
Number of groups =        51                    Obs per group: min =        13
                                                               avg =      17.5
                                                               max =        18
OLS estimation
Estimates efficient for homoskedasticity only
Statistics robust to heteroskedasticity and clustering on sfips
Number of clusters (sfips) = 51                       Number of obs =      891
                                                      F( 20,    50) =   493.12
                                                      Prob > F      =   0.0000
Total (centered) SS     =  22.53257759                Centered R2   =   0.8413
Total (uncentered) SS   =  22.53257759                Uncentered R2 =   0.8413
Residual SS             =  3.575736653                Root MSE      =   .06604
-----------------------------------------------------------------------------------------------
Per capita                    |               Robust
Healthcare expenditure        |      Coef.   Std. Err.      t    P>|t|     [95% Conf. Interval]
-------------+---------------------------------------------------------------------------------
Explanatory Variables Lagged One Period
Prevalence of smoking         |  -.9134923   .0503097   -18.16   0.000    -1.014542   -.8124423
Per capita personal income    |   .0122014   .0974283     0.13   0.901    -.1834891    .2078919
Percent of pop. ≥ age 65      |    .062199   .0765515     0.81   0.420    -.0915591    .2159572
Percent of pop. Hispanic      |  -.0200795     .01178    -1.70   0.094    -.0437404    .0035813
Percent of pop. African-American|  -.0111565   .0073522    -1.52   0.135    -.0259238    .0036109
Cigarette tax. Far West       |  -.1114289   .0194851    -5.72   0.000     -.150566   -.0722919
Cigarette tax. Great Lakes    |   -.107772   .0113739    -9.48   0.000    -.1306172   -.0849268
Cigarette tax. Mideast        |   -.101676   .0281071    -3.62   0.001    -.1581308   -.0452213
Cigarette tax. New England    |  -.0496498   .0099008    -5.01   0.000    -.0695362   -.0297633
Cigarette tax. Plains         |  -.1754729    .020694    -8.48   0.000    -.2170381   -.1339077
Cigarette tax. Rocky Mountains  |  -.0917642   .0098254    -9.34   0.000     -.111499   -.0720293
Cigarette tax. Southeast      |  -.0791882   .0235171    -3.37   0.001    -.1264236   -.0319528
Cigarette tax. Southwest      |  -.1451466   .0231161    -6.28   0.000    -.1915765   -.0987166
National average per capita
healthcare expenditure        |  -.0886295   .1279677    -0.69   0.492    -.3456602    .1684011
Principal component term      |   .4460905   .1900547     2.35   0.023     .0643545    .8278265
Instruments for Cigarette Consumption Per Smoker
Lagged two periods
Prevalence of smoking         |   .6469492    .123839     5.22   0.000     .3982113    .8956872
Cigarette consumption per smoker|   .5671058   .1005024     5.64   0.000     .3652409    .7689707
Percent of pop. ≥ age 65      |  -.0166329   .1049755    -0.16   0.875    -.2274824    .1942166
Lagged three periods
Prevalence of smoking         |   .1752814   .1003151     1.75   0.087    -.0262073    .3767701
Cigarette consumption per smoker|   .1903975   .0787447     2.42   0.019      .032234    .3485609
-----------------------------------------------------------------------------------------------
Included instruments: lls_s lly_s lla_s llhs_s llb_s llt_fwca llt_gl llt_me
                      llt_nema llt_pl llt_rm llt_sete llt_swaz llhr_ue lvc3
                      llls_s lllcps_s llly_s l2ls_s l2lcps
-----------------------------------------------------------------------------------------------
Partial R-squared of excluded instruments:   0.5859
Test of excluded instruments:
  F(  5,    50) =    63.23
  Prob > F      =   0.0000

Summary results for first-stage regressions
-------------------------------------------
Variable    | Shea Partial R2 |   Partial R2    |  F(  5,    50)    P-value
llcps_s     |     0.5859      |     0.5859      |       63.23       0.0000
NB: first-stage F-stat cluster-robust

Underidentification tests
Ho: matrix of reduced form coefficients has rank=K1-1 (underidentified)
Ha: matrix has rank=K1 (identified)
```

```
Kleibergen-Paap rk LM statistic            Chi-sq(5)=23.47    P-val=0.0003
Kleibergen-Paap rk Wald statistic          Chi-sq(5)=329.51   P-val=0.0000

Weak identification test
Ho: equation is weakly identified
Kleibergen-Paap Wald rk F statistic                63.23
See main output for Cragg-Donald weak id test critical values

Weak-instrument-robust inference
Tests of joint significance of endogenous regressors B1 in main equation
Ho: B1=0 and overidentifying restrictions are valid
Anderson-Rubin Wald test     F(5,50)=  8.96      P-val=0.0000
Anderson-Rubin Wald test     Chi-sq(5)=46.69     P-val=0.0000
Stock-Wright LM S statistic  Chi-sq(5)=16.98     P-val=0.0045

NB: Underidentification, weak identification and weak-identification-robust
    test statistics cluster-robust
Number of clusters             N_clust  =         51
Number of observations               N  =        891
Number of regressors                 K  =         16
Number of instruments                L  =         20
Number of excluded instruments       L1 =          5
IV (2SLS) estimation
Estimates efficient for homoskedasticity only
Statistics robust to heteroskedasticity and clustering on sfips

Number of clusters (sfips) = 51                       Number of obs =      891
                                                      F( 16,    50) =   150.25
                                                      Prob > F      =   0.0000
Total (centered) SS     =  8.454356896                Centered R2   =   0.9137
Total (uncentered) SS   =  8.454356896                Uncentered R2 =   0.9137
Residual SS             =  .7299763475                Root MSE      =   .02948
------------------------------------------------------------------------------------------
per capita                      |               Robust
healthcare expenditure          |      Coef.   Std. Err.      z    P>|z|     [95% Conf. Interval]
--------------------------------+---------------------------------------------------------
All Explanatory Variables Lagged One Period
Cigarette consumption per smoker|   .1083824   .0253498     4.28   0.000     .0586978     .158067
Prevalence of smoking           |   .1175569   .0259265     4.53   0.000     .0667419    .1683718
Per capita personal income      |   .2241373   .0674147     3.32   0.001     .0920069    .3562677
Percent of pop. ≥ age 65        |   .5298115   .0936288     5.66   0.000     .3463024    .7133206
Percent of pop. Hispanic        |   .0108264   .0076313     1.42   0.156    -.0041306    .0257835
Percent of pop. African-American|   .0130338   .0063159     2.06   0.039     .0006549    .0254127
Cigarette tax. Far West         |   .0178305   .0312336     0.57   0.568    -.0433863    .0790473
Cigarette tax. Great Lakes      |  -.0066224   .0150612    -0.44   0.660    -.0361419     .022897
Cigarette tax. Mideast          |    .020326    .010637     1.91   0.056    -.0005222    .0411743
Cigarette tax. New England      |   .0477122   .0103496     4.61   0.000     .0274273    .0679971
Cigarette tax. Plains           |   .0357783    .017886     2.00   0.045     .0007225    .0708342
Cigarette tax. Rocky Mountains  |  -.0108379    .013132    -0.83   0.409    -.0365762    .0149004
Cigarette tax. Southeast        |   .0185895   .0229348     0.81   0.418     -.026362    .0635409
Cigarette tax. Southwest        |   5.45e-07   .0247652     0.00   1.000    -.0485384    .0485395
National average per capita
healthcare expenditure          |   .8638085    .095879     9.01   0.000      .675889    1.051728
Principal component term        |  -.5635199   .1317963    -4.28   0.000    -.8218359   -.3052039
------------------------------------------------------------------------------------------
Underidentification test (Kleibergen-Paap rk LM statistic):             23.468
                                                  Chi-sq(5) P-val =    0.0003
------------------------------------------------------------------------------------------
Weak identification test (Kleibergen-Paap rk Wald F statistic):         63.231
Stock-Yogo weak ID test critical values:  5% maximal IV relative bias    18.37
                                         10% maximal IV relative bias    10.83
                                         20% maximal IV relative bias     6.77
                                         30% maximal IV relative bias     5.25
                                         10% maximal IV size             26.87
                                         15% maximal IV size             15.09
                                         20% maximal IV size             10.98
                                         25% maximal IV size              8.84
Source: Stock-Yogo (2005).  Reproduced by permission.
NB: Critical values are for Cragg-Donald F statistic and i.i.d. errors.
------------------------------------------------------------------------------------------
Hansen J statistic (overidentification test of all instruments):        12.112
```

```
                                                   Chi-sq(4) P-val =    0.0165
------------------------------------------------------------------------------
Instrumented:         llcps_s
Included instruments: lls_s lly_s lla_s llhs_s llb_s llt_fwca llt_gl llt_me
                      llt_nema llt_pl llt_rm llt_sete llt_swaz llhr_ue lvc3
Excluded instruments: llls_s lllcps_s llly_s l2ls_s l2lcps
-------------------------------------------------------------------------------------------------
```

Table S4. Results of sensitivity analysis of regression on states with low, mid, and high serial correlation in the panel data regression residuals using the Model in Table 1, main text			
	Tertiles		
Level of autocorrelation in panel data regression residuals	Low third<br>(-0.18 to 0.52)	Mid Third<br>(0.52 to 0.67)	High Third<br>(0.67 to 0.75)
Coefficient (SE)			
Prevalence of cigarette smoking	0.146<br>(0.0356)	0.0998<br>(0.0275)	0.209<br>(0.0576)
Cigarette consumption per smoker	0.143<br>(0.0443)	0.0677<br>(0.0187)	0.260<br>(0.0481)
Statistics for instrumental variables tests			
Underidentification test (Kleibergen-Paap rk LM statistic)	26.8<br>(P = 0.0001)	58.7<br>(P < 0.0001)	40.0<br>(P < 0.0001)
Weak identification test (Kleibergen-Paap rk Wald F statistic)	7.292	107	26.78
Hansen J statistic (overidentification test of all instruments)	3.52<br>(P=0.475)	5.26<br>(P=0.262)	12.21<br>(P=0.0159)

Table S5. Final Regression results, CMS state resident healthcare expenditure, 1992-2009 after specification search for additional lags in explanatory variables (Compare to Table 1 in main text)

```
IV (2SLS) estimation
Estimates efficient for homoskedasticity only
Statistics robust to heteroskedasticity and clustering on sfips
Number of clusters (sfips) = 51                     Number of obs =      891
                                                    F( 18,    50) =   149.52
                                                    Prob > F      =   0.0000
Total (centered) SS     =  8.454356896              Centered R2   =   0.9132
Total (uncentered) SS   =  8.454356896              Uncentered R2 =   0.9132
Residual SS             =  .7339810469              Root MSE      =   .02956

Dependent variable ln(per capita health care expenditures)
All explanatory variables are natural logarithms
-----------------------------------------------------------------------------------------------
  per capita                 |                Robust
  healthcare expenditure     |      Coef.   Std. Err.      z    P>|z|     [95% Conf. Interval]
-------------+---------------------------------------------------------------------------------
Explanatory variables lagged one period
Cig. consumption per smoker  |   .1378927   .0279701     4.93   0.000     .0830723     .192713
Prevalence of smoking        |   .1400058   .0258194     5.42   0.000     .0894007     .190611
Per capita personal income   |   .0540762   .0538141     1.00   0.315    -.0513975    .1595498
Percent pop. > age 65        |   .5302044   .0906563     5.85   0.000     .3525214    .7078874
Percent pop. Hispanic        |   .0123086   .0077966     1.58   0.114    -.0029723    .0275896
percent pop. African-American |  .0127462    .005681     2.24   0.025     .0016118    .0238807
Cigarette tax, Far West      |   .0353853   .0293113     1.21   0.227    -.0220638    .0928344
Cigarette tax, Rocky Mountains| -.0113605   .0129094    -0.88   0.379    -.0366624    .0139414
Cigarette tax, Southwest     |   .0101097   .0228078     0.44   0.658    -.0345929    .0548122
Cigarette tax, Southeast     |   .0193253   .0226781     0.85   0.394    -.0251229    .0637736
Cigarette tax, Plains        |   .0413251   .0184614     2.24   0.025     .0051416    .0775087
Cigarette tax, Great Lakes   |  -.0004015   .0147746    -0.03   0.978    -.0293591    .0285561
Cigarette tax, Mideast       |    .027177   .0115027     2.36   0.018     .0046322    .0497218
Cigarette tax, New England   |   .0491126   .0106023     4.63   0.000     .0283326    .0698926
National average per capita
healthcare expenditure       |   .8399335   .1091038     7.70   0.000     .6260939    1.053773
Principal component term     |  -.5278268   .1335797    -3.95   0.000    -.7896382   -.2660155
Explanatory variables lagged two periods
Per capita personal income   |   .2242859   .1036033     2.16   0.030     .0212272    .4273446
Percent pop. African-American |  .0128128   .0057258     2.24   0.025     .0015903    .0240352
-----------------------------------------------------------------------------------------------
Underidentification test (Kleibergen-Paap rk LM statistic):            22.691
                                                   Chi-sq(3) P-val =    0.0000
-----------------------------------------------------------------------------------------------
Weak identification test (Kleibergen-Paap rk Wald F statistic):        43.977
Stock-Yogo weak ID test critical values:  5% maximal IV relative bias    13.91
                                         10% maximal IV relative bias     9.08
                                         20% maximal IV relative bias     6.46
                                         30% maximal IV relative bias     5.39
                                         10% maximal IV size             22.30
                                         15% maximal IV size             12.83
                                         20% maximal IV size              9.54
                                         25% maximal IV size              7.80
Source: Stock-Yogo (2005).  Reproduced by permission.
NB: Critical values are for Cragg-Donald F statistic and i.i.d. errors.
-----------------------------------------------------------------------------------------------
Hansen J statistic (overidentification test of all instruments):        2.108
                                                   Chi-sq(2) P-val =    0.3485
-------------------------------------------------------------------------------
Instrumented:         llcps_s
Included instruments: lls_s lly_s lla_s llhs_s llb_s llt_fwca llt_rm llt_swaz
                      llt_sete llt_pl llt_gl llt_me llt_nema llhr_ue lvc3 llly_s
                      lllb_s
Excluded instruments: llls_s l2ls_s l2lcps
-----------------------------------------------------------------------------------------------
```

Table S6. Final Regression results, CMS state resident healthcare expenditure, 1992-2009, prevalence instrumenting prevalence of smoking and cigarette consumption per smoker with irrelevant instrumental variables (Compare to Table 1 in main text)

```
IV (2SLS) estimation
--------------------
Estimates efficient for homoskedasticity only
Statistics robust to heteroskedasticity and clustering on sfips
Number of clusters (sfips) = 51                  Number of obs =      911
                                                 F( 16,    50) =   217.59
                                                 Prob > F      =   0.0000
Total (centered) SS     =  8.922263544           Centered R2   =   0.9133
Total (uncentered) SS   =  8.922263544           Uncentered R2 =   0.9133
Residual SS             =  .7734256891           Root MSE      =   .02999

Dependent variable ln(per capita health care expenditures)
All explanatory variables are natural logarithms and lagged one peiod
------------------------------------------------------------------------------------------
Per capita                    |               Robust
Healthcare expenditure        |      Coef.   Std. Err.      z    P>|z|     [95% Conf. Interval]
-------------+------------------------------------------------------------------------------
All Explanatory Variables Lagged One Period
Cig. consumption per smoker   |   .0849149   .0931946     0.91   0.362    -.0977432    .2675729
Prevalence of smoking         |   .1699522   .0702055     2.42   0.015      .032352    .3075524
Per capita personal income    |   .2341345   .0849729     2.76   0.006     .0675907    .4006783
Percent pop. > age 65         |   .5507732   .0892833     6.17   0.000     .3757811    .7257653
Percent pop. Hispanic         |   .0097585   .0088257     1.11   0.269    -.0075394    .0270565
percent pop. African-American |   .0122438   .0076005     1.61   0.107    -.0026529    .0271406
Cigarette tax, Far West       |   .0181194   .0310352     0.58   0.559    -.0427085    .0789473
Cigarette tax, Rocky Mountains|  -.0143127   .0187949    -0.76   0.446    -.0511501    .0225247
Cigarette tax, Southwest      |  -.0104237   .0320467    -0.33   0.745     -.073234    .0523867
Cigarette tax, Southeast      |   .0156407   .0240384     0.65   0.515    -.0314738    .0627552
Cigarette tax, Plains         |   .0335947   .0253407     1.33   0.185    -.0160721    .0832616
Cigarette tax, Great Lakes    |  -.0094695   .0280904    -0.34   0.736    -.0645257    .0455867
Cigarette tax, Mideast        |   .0186608    .029083     0.64   0.521    -.0383407    .0756624
Cigarette tax. New England    |   .0494299    .016731     2.95   0.003     .0166378     .082222
National average per capita
healthcare expenditure        |   .8713927   .0615277    14.16   0.000     .7508005    .9919848
Principal component term      |  -.4467698   .1136064    -3.93   0.000    -.6694343   -.2241053
------------------------------------------------------------------------------------------
Underidentification test (Kleibergen-Paap rk LM statistic):             29.945
                                                   Chi-sq(13) P-val =   0.0048
------------------------------------------------------------------------------------------
Weak identification test (Kleibergen-Paap rk Wald F statistic):          5.349
Stock-Yogo weak ID test critical values:  5% maximal IV relative bias    19.83
                                         10% maximal IV relative bias    10.89
                                         20% maximal IV relative bias     6.20
                                         30% maximal IV relative bias     4.53
                                         10% maximal IV size             36.36
                                         15% maximal IV size             19.72
                                         20% maximal IV size             14.05
                                         25% maximal IV size             11.13
Source: Stock-Yogo (2005).  Reproduced by permission.
NB: Critical values are for Cragg-Donald F statistic and i.i.d. errors.
------------------------------------------------------------------------------------------
Hansen J statistic (overidentification test of all instruments):        38.210
                                                   Chi-sq(12) P-val =   0.0001
------------------------------------------------------------------------------------------
Instrumented:         lls_s llcps_s
Included instruments: lly_s lla_s llhs_s llb_s llt_fwca llt_rm llt_swaz llt_sete
                      llt_pl llt_gl llt_me llt_nema llhr_ue lvc3
Excluded instruments: ic1 ic2 ic3 ic4 ic5 ic6 ic7 ic8 ic9 ic10 ic11 ic12 ic13 ic14
------------------------------------------------------------------------------------------
```

Table S7. Average Excess Expenditures due to Departures of Regional Smoking Behavior and Cigarette Taxes from National Average (State population weighted), 1992 - 2009								
BEA Region	New England NE	Mideast ME	Great Lakes GL	Plains PL	Southeast SE	Southwest SW	Rocky Mountain RM	Far West FW
Attributable to prevalence of smoking ($\$_{2010}$ per capita)								
Mean	-394	-34.7	62.7	-21.7	66.4	-6.53	-119	-34.5
SE	87.1	7.65	13.9	4.74	14.6	1.45	26.2	7.62
Attributable to mean cigarette consumption per smoker ($\$_{2010}$ per capita)								
Mean	-38.9	-150	-35.5	31.5	77.9	-137	-48.2	-210
SE	9.07	35.0	8.34	7.34	18.2	32.0	11.2	49.2
Attributable to differences in smoking behavior: prevalence and mean cigarette consumption per smoker ($\$_{2010}$ per capita)								
Mean	-433	-185	27.2	9.84	144	-144	-167	-245
SE	92.1	39.5	11.9	6.41	28.6	32.8	33.3	53.5
Attributable to state tax differential effects ($\$_{2010}$ per capita)								
Mean	114	44.4	-3.54	-41.9	-58.6	-0.00218	15.0	20.5
SE	24.6	23.2	7.96	21.1	72.5	0.538	18.1	35.7
Implied proportional difference between measured and true cigarette consumption per smoker ($\$_{2010}$ per capita)								
Mean	0.523	0.222	-0.019	-0.17	-0.238	0.00000896	0.0938	0.135
SE	0.113	0.116	0.0427	0.0853	0.295	0.00221	0.113	0.235
Total attributable to differences in smoking behavior including state tax differential effects ($\$_{2010}$ per capita)								
Mean	-320	-141	23.7	-32.1	85.7	-144	-152	-224
SE	94.9	11.6	44.3	20.2	87.5	32.9	38.5	50.8
Total regional difference, including state tax differential effects ($\$_{2010}$ million)								
Mean	-4620	-6750	-6750	-652	6660	-5330	-1650	-11700
SE	1370	2130	1370	410	6800	1220	419	2650

Note: Negative dollar amounts indicate savings, positive dollar amounts indicate excess expenditures compared to national average smoking behavior. Negative proportions indicate estimated true consumption is less than measured consumption, positive proportions indicate estimated true consumption is less than measured consumption. Bureau of Economic Analysis Regions, NE: New England, ME: Mideast, GL: Great Lakes, PL: Plains, SE: Southeast, SW: Southwest, RM: Rocky Mountains, FW: Far West.

Table S8. Average Excess Expenditures due to Departures of State Smoking Behavior and Cigarette Taxes from National Average, 1992 – 2009

State	A: Current smoking: Prevalence ($ per capita per year)		B: Mean consumption per smoker ($ per capita per year)		C: Smoking behavior unadjusted for unmeasured consumption due to state tax differentials ($ per capita per year) (Cols A+B)		D: Adjustment for unmeasured consumption due to state tax differentials ($ per capita per year)		E: Proportion of measured consumption that is due to state tax differentials		F: Smoking behavior adjusted for unmeasured consumption ($ per capita per year) (Col C + D)		G: Total state smoking behavior, adjusted to unmeasured consumption for year 2009 ($ million per year) (Col F x state population)	
AK	115	(25)	-189	(44)	-75	(45)	36	(63)	0.16	(0.28)	-39	(65)	-27	(46)
AL	34	(7)	68	(16)	102	(19)	-52	(64)	-0.22	(0.27)	49	(74)	233	(349)
AR	95	(21)	22	(5)	118	(23)	-22	(27)	-0.09	(0.11)	96	(42)	277	(122)
AZ	-55	(12)	-189	(44)	-244	(49)	0	(36)	0.00	(0.24)	-244	(59)	-1610	(391)
CA	-201	(44)	-229	(54)	-431	(77)	15	(26)	0.11	(0.19)	-416	(79)	-15376	(2920)
CO	-35	(8)	-67	(16)	-102	(19)	21	(25)	0.12	(0.14)	-81	(36)	-408	(182)
CT	-92	(20)	-61	(14)	-153	(27)	116	(25)	0.53	(0.12)	-37	(37)	-131	(130)
DC	-102	(23)	-230	(54)	-333	(63)	34	(18)	0.18	(0.1)	-299	(69)	-179	(41)
DE	51	(11)	349	(82)	399	(85)	-24	(12)	-0.05	(0.03)	375	(88)	332	(78)
FL	-12	(3)	20	(5)	8	(5)	-30	(36)	-0.14	(0.17)	-22	(37)	-399	(688)
GA	-18	(4)	58	(14)	40	(13)	-60	(74)	-0.29	(0.36)	-20	(77)	-199	(762)
HI	-128	(28)	-289	(68)	-417	(79)	50	(87)	0.33	(0.57)	-368	(96)	-477	(124)
IA	-19	(4)	52	(12)	34	(12)	-23	(12)	-0.10	(0.05)	10	(13)	30	(39)
ID	-98	(22)	-17	(4)	-116	(23)	18	(22)	0.11	(0.13)	-98	(32)	-151	(49)
IL	18	(4)	-126	(30)	-109	(29)	-5	(11)	-0.03	(0.06)	-114	(29)	-1467	(371)
IN	123	(27)	130	(30)	253	(45)	12	(27)	0.04	(0.08)	265	(59)	1702	(377)
KS	-56	(12)	-27	(6)	-83	(15)	-32	(16)	-0.17	(0.09)	-115	(26)	-325	(74)
KY	209	(46)	278	(65)	487	(88)	-88	(109)	-0.22	(0.27)	399	(169)	1723	(731)
LA	58	(13)	53	(12)	111	(20)	-45	(56)	-0.20	(0.24)	66	(67)	295	(301)
MA	-86	(19)	-134	(31)	-220	(40)	132	(29)	0.67	(0.15)	-88	(48)	-577	(317)
MD	-81	(18)	-96	(23)	-177	(32)	20	(11)	0.09	(0.05)	-157	(36)	-895	(204)
ME	21	(5)	5	(1)	26	(5)	112	(24)	0.39	(0.09)	138	(25)	182	(33)
MI	72	(16)	-100	(23)	-28	(25)	-19	(43)	-0.08	(0.19)	-47	(42)	-470	(415)
MN	-67	(15)	-18	(4)	-85	(16)	34	(17)	0.18	(0.09)	-50	(21)	-264	(110)
MO	105	(23)	116	(27)	220	(40)	-131	(65)	-0.46	(0.23)	90	(65)	537	(389)
MS	59	(13)	67	(16)	126	(23)	-63	(77)	-0.27	(0.33)	63	(89)	186	(264)
MT	-69	(15)	-3	(1)	-72	(15)	2	(2)	0.01	(0.01)	-71	(16)	-69	(15)
NC	79	(17)	111	(26)	190	(34)	-84	(103)	-0.32	(0.39)	106	(122)	994	(1143)
ND	-44	(10)	-30	(7)	-74	(13)	-33	(16)	-0.16	(0.08)	-107	(25)	-69	(16)
NE	-49	(11)	-7	(2)	-57	(11)	-29	(15)	-0.15	(0.07)	-86	(21)	-154	(37)

Table S8. Average Excess Expenditures due to Departures of State Smoking Behavior and Cigarette Taxes from National Average, 1992 – 2009 (Cont.)														
	A		B		C		D		E		F		G	
State	Current smoking: Prevalence ($ per capita per year)		Mean consumption per smoker ($ per capita per year)		Smoking behavior unadjusted for unmeasured consumption due to state tax differentials ($ per capita per year) (Cols A+B)		Adjustment for unmeasured consumption due to state tax differentials ($ per capita per year)		Proportion of measured consumption that is due to state tax differentials		Smoking behavior adjusted for unmeasured consumption ($ per capita per year) (Col C + D)		Total state smoking behavior, adjusted to unmeasured consumption for year 2009 ($ million per year) (Col F x state population)	
NH	-8	(2)	397	(93)	389	(92)	-9	(2)	-0.02	(0.004)	380	(91)	503	(121)
NJ	-94	(21)	-124	(29)	-218	(39)	73	(38)	0.36	(0.19)	-145	(55)	-1263	(483)
NM	-34	(8)	-256	(60)	-290	(62)	0	(28)	0.00	(0.19)	-290	(74)	-583	(148)
NV	132	(29)	-81	(19)	52	(31)	-8	(14)	-0.03	(0.05)	44	(29)	115	(76)
NY	-25	(5)	-269	(63)	-294	(64)	59	(31)	0.33	(0.17)	-235	(71)	-4595	(1382)
OH	87	(19)	24	(6)	112	(21)	3	(8)	0.01	(0.03)	115	(25)	1327	(292)
OK	89	(20)	50	(12)	139	(25)	0	(30)	0.00	(0.11)	139	(43)	513	(158)
OR	-66	(15)	-4	(1)	-71	(15)	24	(42)	0.10	(0.17)	-47	(40)	-179	(153)
PA	43	(10)	-40	(9)	4	(12)	18	(10)	0.07	(0.03)	22	(13)	279	(169)
RI	-7	(2)	-59	(14)	-66	(14)	172	(37)	0.69	(0.15)	106	(37)	112	(39)
SC	58	(13)	110	(26)	168	(31)	-91	(112)	-0.35	(0.43)	77	(128)	352	(583)
SD	-21	(5)	-10	(2)	-31	(6)	-23	(12)	-0.12	(0.06)	-54	(15)	-44	(12)
TN	112	(25)	81	(19)	193	(35)	-60	(74)	-0.23	(0.28)	134	(95)	842	(595)
TX	-26	(6)	-142	(33)	-167	(35)	0	(4)	0.00	(0.02)	-167	(37)	-4145	(918)
UT	-398	(88)	-72	(17)	-470	(93)	5	(6)	0.05	(0.06)	-465	(97)	-1295	(271)
VA	-12	(3)	116	(27)	104	(27)	-87	(107)	-0.37	(0.45)	17	(117)	133	(920)
VT	-48	(11)	105	(25)	57	(24)	69	(15)	0.23	(0.05)	125	(29)	78	(18)
WA	-58	(13)	-260	(61)	-319	(65)	53	(93)	0.32	(0.57)	-265	(92)	-1769	(616)
WI	13	(3)	-23	(5)	-10	(5)	-5	(11)	-0.02	(0.05)	-15	(10)	-84	(59)
WV	135	(30)	67	(16)	202	(37)	-41	(51)	-0.15	(0.18)	161	(75)	293	(137)
WY	7	(2)	75	(18)	83	(18)	28	(34)	0.12	(0.14)	111	(32)	60	(18)

Table S9. Variation in prevalence of current smoking and cigarette consumption per smoker, 1992-2009				
Percentile	Absolute value of proportional change around individual state means		Absolute value of proportional annual change	
	Prevalence of current cigarette smoking ($s_{i,t}$)	Cigarette consumption per current smoker ($cps_{i,t}$)	Prevalence of current cigarette smoking ($s_{i,t}$)	Cigarette consumption per current smoker ($cps_{i,t}$)
5%	.00409	.00850	.00451	.00444
10%	.0101	.0209	.00985	.0100
25%	.0252	.0534	.0232	.0265
50%	.0526	.109	.0471	.0584
75%	.108	.190	.0821	.103
90%	.169	.268	.119	.156
95%	.209	.322	.138	.207
